# Alleviating NIR-II emission quenching in ring-fused fluorophore via manipulating dimer populations for superior fluorescence imaging

**DOI:** 10.1038/s41377-025-01787-0

**Published:** 2025-03-04

**Authors:** Xiaofei Miao, Mingxuan Jia, Xianwei Weng, Jie Zhang, Yonghui Pan, Hui Zhao, Zhongzheng Yu, Quli Fan, Wenbo Hu

**Affiliations:** 1 State Key Laboratory of Flexible Electronics (LoFE) & Institute of Advanced Materials (IAM), Nanjing University of Posts & Telecommunications, Nanjing, China; 2 State Key Laboratory of Flexible Electronics (LoFE) & Institute of Flexible Electronics (IFE), Northwestern Polytechnical University, Xi’an, China; 3 Cavendish Laboratory, University of Cambridge, Cambridge, UK

**Keywords:** Biophotonics, Near-infrared spectroscopy

## Abstract

Emission quenching resulting from fluorophore aggregation has long been a significant challenge in optimizing emission-based technologies, such as fluorescence imaging and optoelectronic devices. Alleviating this quenching in aggregates is crucial, yet progress is impeded by the limited understanding of the nature and impact of aggregates on emission. Here, we elucidate the critical role of dimeric aggregate (dimer) in alleviating second near-infrared (NIR-II, 900-1700 nm) emission quenching from ring-fused fluorophore 4F for superior fluorescence imaging. Spectral decomposition and molecular dynamics simulations demonstrate the predominance of dimer populations in 4F aggregates. Notably, dimers exhibit significantly weaker emission but intense intermolecular nonradiative (*inter*NR) decay compared to monomers, as demonstrated by ultrafast spectra and quantum calculation. Therefore, the predominant population of dimers with weak emission and pronounced *inter*NR feature underlies the emission quenching in 4F aggregates. This discovery guides the preparation of ultrabright NIR-II 4F nanofluorophore (4F NP3s) by decreasing dimer populations, which show 5-fold greater NIR-II brightness than indocyanine green, enabling superior resolution in visualizing blood vessels. This work offers valuable insights into aggregation-caused quenching, with broad implications extending far beyond NIR-II fluorescence imaging.

## Abstract (web summary)

This study presents a dimer-dominated emission quenching mechanism that enhances second near-infrared emission in ring-fused fluorophores by reducing dimer populations for high-resolution vascular fluorescence imaging.

## Graphical abstract

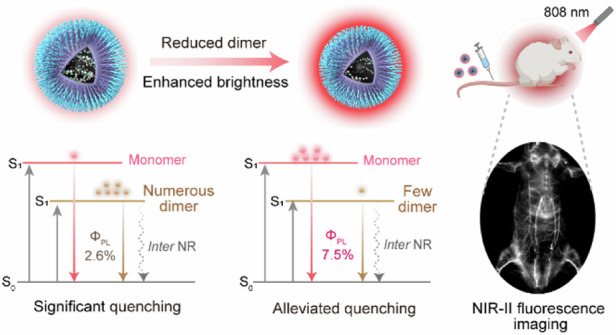

## Introduction

Fluorescence imaging in the second near-infrared window (NIR-II, 900–1700 nm) holds great promise for advancing both basic research and clinical diagnostics due to its ability to provide high-quality images of deep tissue structures with minimal invasiveness^1–6^. Achieving high-quality imaging requires fluorophores with high fluorescence brightness, which is defined by the product of the extinction coefficient (ε) and photoluminescence quantum yield (Φ_PL_) in the NIR-II window^7,8^. To develop bright NIR-II fluorophores, two primary strategies have been established by donor-acceptor (D-A) engineering and conjugation extension^9–15^. However, these NIR-II fluorophores are prone to aggregation-caused quenching (ACQ) in practical applications^16–19^, which typically reduces Φ_PL_ values to below 2% (Table S1). Worse still, D-A engineering leads to weak absorption in the long wavelengths^20^. In contrast, conjugation extension can improve long-wavelength absorption but often shows ultralow Φ_PL_ due to more pronounced ACQ and molecular motion^21^. These limitations result in unsatisfactory NIR-II brightness for most available NIR-II fluorophores, with values typically below 1000 M^−1^ cm^−1^ (Table S1). Therefore, there remains an urgent need to develop bright NIR-II fluorophores.

Alleviating ACQ while achieving intense absorption is a promising strategy for developing bright NIR-II fluorophore. ACQ typically arises from fluorophore aggregation, which brings molecules into close contact, facilitating nonradiative energy loss via intermolecular interactions. To mitigate ACQ, several strategies have been employed, including the introduction of bulky substitutes into traditional D-A NIR-II fluorophores to extend intermolecular distance^22–24^, or designing aggregation-induced emission fluorogens (AIEgens) with twisted and rotation-flexible segments to counteract ACQ^25–28^. Although these strategies considerably alleviate ACQ, traditional NIR-II fluorophores used in these designs exhibit weak absorption due to disrupted conjugation, ultimately result in suboptimal NIR-II fluorescence brightness^29,30^. Ring-fused fluorophores, which typically display intense absorption and emission, have recently gained significant attention as novel NIR-II fluorophores (Table S2). However, these ring-fused NIR-II fluorophores also undergo severe ACQ, showing a dramatic decrease in Φ_PL_ from 15.1% in unimolecular state to 2.1% in practical application (aggregated state)^31^. Thus, further improvement in NIR-II brightness is principally achievable if ACQ can be significantly alleviated. Given the importance of aggregates in ACQ, elucidating their nature and precise role on emission is of both fundamental and practical significance in alleviating ACQ. Unfortunately, the exact nature and role of these aggregates remain poorly understood.

**Fig. 1:**
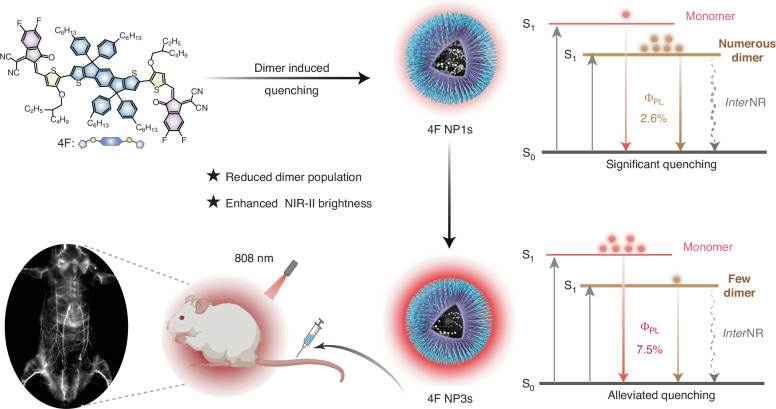
Schematic diagram of the remarkable alleviation of NIR-II emission quenching in ring-fused fluorophore via manipulating dimer population for superior fluorescence imaging in vivo. *PL* photoluminescence, *interNR* intermolecular nonradiative

Herein, we demonstrate the nature and critical role of dimer in alleviating NIR-II emission quenching in fluorophore aggregates (Fig. 1). The hydrophobic ring-fused fluorophore (4F) was designed and encapsulated into an amphiphilic copolymer (Pluronic F-127) to create water-soluble nanoparticles (4F NP1s), through which we investigate the nature and role of aggregates in modulating NIR-II emission brightness. Spectral decomposition and molecular dynamics calculations reveal that both dimers and monomers contribute to the NIR-II emission within 4F NP1s, with dimers populations predominating over monomers. Femtosecond transient absorption (fs-TA) experiments and quantum chemical calculations elucidate the weakly emissive and efficient intermolecular non-radiative (*inter*NR) decay characteristics of dimers, in contrast to the emissive monomers. Together, the predominant population of dimer with weak emission and intense *inter*NR nature underscores the ACQ observed in 4F NP1s. Based on these findings, we alleviate ACQ by decreasing dimer population through a simple aggregation control strategy. This produces an ultrabright 4F aggregate (4F NP3s) with brightness of 7.1 × 10³ M^−1^ cm^−1^, which is nearly 5-times greater than that of indocyanine green (ICG) at 1560 M^−1^ cm^-1^ (Figs. S1, 2), repressing the top-level NIR-II fluorescence brightness. These properties enable 4F NP3s to provide superior resolution in visualizing blood vessels.

## Results

### Design and characterization

The synthesis of 4F was achieved using a ring-fused donor based on the 1,2-dimethyl-1H-core of (4,4,9,9-tetrakis (4-hexylphenyl) -4,9-dihydro-s-indaceno [1,2-b:5,6-b’] dithiophene-2,7-diyl) bis (trimethylstannane) (Scheme S1). A strong acceptor, 2-(5,6-difluoro-3-oxo-2,3-dihydro-1H-inden-1-ylidene) malononitrile, was linked to the ring-fused donor via a thiophene linker with alkoxy chains. This extensive π-conjugated structure was designed to produce a narrowed bandgap and delocalized electrons, aiming for intense long-wavelength absorption and emission. The molecular structure of 4F was confirmed by ^1^H NMR, ^13^C NMR, and mass spectrometry (Fig. S3–S8).

### Basic optical properties

**Fig. 2:**
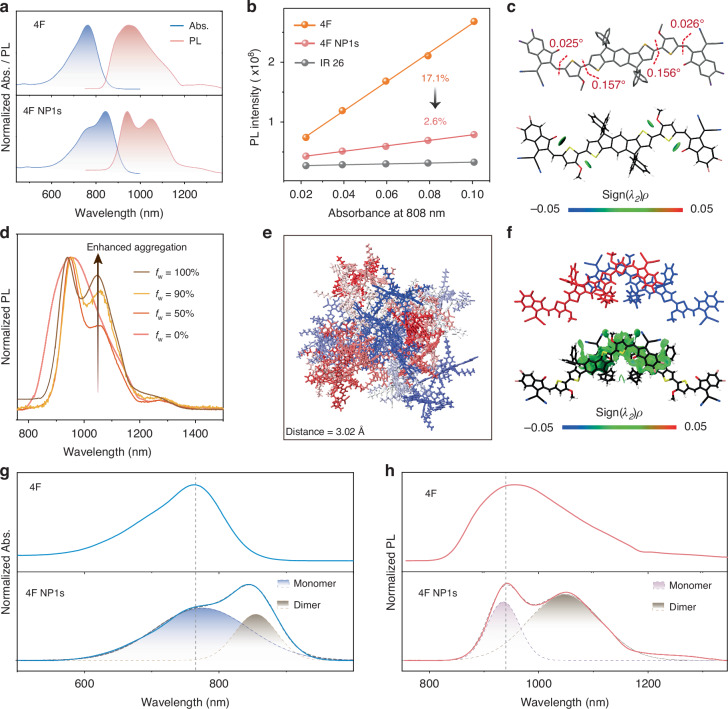
Optical properties of 4F in unimolecular and aggregated states. **a** Absorption (Abs.) and PL spectra of 4F in THF and 4F NP1s in aqueous solution. **b** Φ_PL_ of 4F and 4F NP1s. **c** Optimized molecular conformation and calculated non-covalent interactions at S_1_ states of 4F. Blue: strong attraction; Green: van der Waals interaction; Red: strong repulsion. **d** Normalized PL spectra of 4F with different degrees of aggregation. The increased fraction of water (*f*_*w*_) in THF/H_2_O mixed solutions suggests enhanced aggregation of 4F (*f*_w_ = 0% corresponds to 4F in pure THF, while *f*_w_ = 100% represents the 4F NP1s). **e** Molecular dynamics simulation snapshot of 4F aggregates in H_2_O (Interfacial distance between dimer: 3.02 Å). **f** Optimized dimer conformation and non-covalent interactions at S_1_ states of 4F. **g**, **h** Decomposition of absorption and PL spectra after Gaussian fitting

In tetrahydrofuran (THF) solution, 4F exhibits robust NIR absorption (ε_808 nm_ = 6.4 × 10^4^ M^−1^ cm^−1^, Fig. 2a, Fig. S9) and intense NIR-II emission with ultrahigh Φ_PL_ of up to 17.1% (reference: IR-26, Φ_PL_ = 0.5%, Fig. 2b, Fig. S10). This performance surpasses that of most reported NIR-II fluorophores^7,10^. The intense long-wavelength absorption and emission arise from the extensive π-conjugation in the fully planar backbone of 4F, with negligible torsion (<0.4˚) in both S_0_ and S_1_ states (Fig. 2c, Fig. S12). Furthermore, natural transition orbitals (NTO) demonstrate the strong electronic couplings (π-conjugation) within 4F, as evidenced by the broad distribution of hole and electron wavefunctions across the entire skeleton with large spatial overlaps O_*h/e*_ (Fig. S13)^32^. This is consistent with the minimal solvation effect (Fig. S14), indicating that the absorption and emission of 4F originate primarily from its extensive π-conjugation, rather than from the conventional charge-transfer (CT) state typically observed in traditional D-A NIR-II fluorophores^33^. The interaction region indicator attributes the planar architecture of 4F to an intramolecular noncovalent S···O conformational lock (Fig. 2c)^34–36^. Notably, this distinctive conformational lock not only supports the planar conformation necessary for intense long-wavelength absorption and emission but also induces a rigid structure that significantly inhibits nonradiative energy loss through molecular motions.

For bioapplications, hydrophobic 4F (1 mg mL^−1^) was encapsulated into Pluronic F-127 to create water-soluble nanoparticles (4F NP1s). Dynamic light scattering (DLS) and transmission electron microscopy (TEM) confirmed that the resulting nanoparticles were uniform, with a hydrodynamic diameter of 50 nm (Fig. S15). Compared to unimolecular 4 F in THF, 4F NP1s exhibited a pronounced redshift in their absorption maximum and an increased ε at 808 nm of 6.8 × 10^4^ M^−1^ cm^−1^ (Figs. 2a, S9). This shift brings the absorption closer to the biologically transparent wavelength around 800 nm, enhancing its potential for bioapplications in deep-seated regions. In contrast, 4F NP1s show a significant decrease in fluorescence, with Φ_PL_ dramatically decreasing from 17.1% to 2.6% (Figs. 2b, S11, Table S3). Correspondingly, the fluorescence brightness of 4F NP1s decreased by an order of magnitude compared to unimolecular 4F in THF (1.79 × 10^3^ M^−1^ cm^−1^ vs. 1.09 × 10^4^ M^−1^cm^−1^), substantially reducing their potential of bioimaging applications.

### Dimer-associated absorption and emission

To investigate the impact of aggregation on optical properties, the spectroscopic properties of 4F with different degrees of aggregation were analyzed. When the fraction of water (*f*_w_) in THF/H_2_O mixed solutions was ≤10%, both the absorption and PL spectra exhibited a similar lineshape to that of 4F in pure THF, indicating a unimolecular nature (Fig. S16). However, upon increasing the *f*_w_ to 20%, 4F displayed a new, red-shifted absorption peak around 850 nm and a PL peak around 1050 nm, corresponding well to the spectral features of 4F NP1s (aggregates). As the *f*_w_ continued to increase, the aggregation-associated emission around 1050 nm became more pronounced, while the overall PL intensity of 4F gradually decreased (Figs. 2d, S16). Molecular dynamics simulation confirmed the coexistence of multiple dimer architectures alongside monomers (Fig. 2e, f; Fig. S17). We thus assign the longer-wavelength absorption of around 850 nm and emission of around 1050 nm to the dimer. Spectral decomposition further reveals the bi-component fluorescence from 4F NP1s (Fig. 2h), with distinct emission peaks corresponding to monomers (~940 nm) and dimers (~1050 nm). Notably, the weight of dimers significantly surpasses that of monomers (64.3% vs. 35.7%) in 4F NP1s, indicating a predominant population of dimers within 4F NP1s (Fig. 2h). Furthermore, quantum chemical calculations predicted that dimers exhibit weaker emissive properties compared to monomers, as indicated by their smaller oscillator strengths (Table S4). Moreover, the close intermolecular distance (~3Å) of dimers within NPs facilitates the intense π-π interaction (Figs. 2f; S17), indicating strong nonradiative decay. Together, these results suggest that the reduced emission observed in 4F aggregates is primarily due to the predominant population of dimers with weak emissive properties.

### Dimer with weak emission and intense intermolecular nonradiative decay

**Fig. 3:**
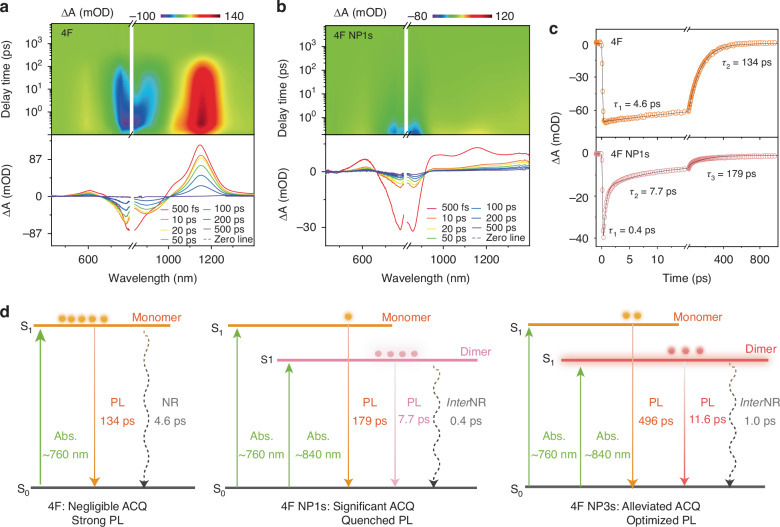
Excited state dynamics underlying emission quenching in 4F aggregates. **a**, **b** Pseudocolor fs-TA mapping of 4F and 4F NP1s pumped at 808 nm laser pulse, respectively. **c** Kinetics traces and fitting lines of 4F and 4F NP1s at the representative wavelength within the ground state bleaching region (4F@766 nm, 4 F NP1s @762 nm). **d** Schematic illustration of the significant alleviation of ACQ by manipulating the dimer population

Femtosecond transient absorption (fs-TA) spectra provided experimental evidence of the weak emission and intense *inter*NR decay from dimers in 4F NP1s^37,38^. As shown in Fig. 3a, b, the fs-TA mapping of 4F and 4F NP1s exhibits prominent ground state bleaching (GSB) signals around 750 nm, where 4F NP1s present accelerated excited state decay compared to 4F in THF, consistent with its reduced emission. The representative kinetic curves within the GSB region around 765 nm reveal three components with time constants of 0.43, 7.73, and 179 ps for 4F NP1s, compared to two components of 4.6 and 134 ps for 4F in THF (Fig. 3c, Table. S5). The 4.6 ps component in 4F is ascribed to intramolecular NR decay. The long-lived component of 134 ps for 4F and 179 ps for 4F NPs are attributed to monomer fluorescence, due to their consistency with the fluorescence lifetime (vide infra).

The femtosecond component of 0.43 ps observed in 4F NP1s corresponds to *inter*NR decay from dimers, while the 7.7 ps component is attributed to dimer emission. This assignment of femtosecond component is supported by the kinetic curves of 4F in various diluted THF solutions (Fig. S18). In principle, a femtosecond component could arise from either *inter*NR decay or structural relaxation. In diluted solutions, 4F favors structural relaxation but disfavors *inter*NR decay. Therefore, the absence of the femtosecond component in these solutions rules out the possibility of structural relaxation, reinforcing the assignment of femtosecond component to dimer. Notably, this femtosecond component suggests a deactivation preference over all other decay pathways within 4F NP1s, highlighting an intense *inter*NR decay that reduces emission^39,40^. In addition, the accelerated radiative decay of dimers (7.7 ps), occurring at a rate nearly 30 times faster than that of monomers (179 ps), also suggests a deactivation preference in radiative pathways for dimers over monomers. This weak dimer emission, but having nonradiative deactivation priority over monomer, further reduces emission.

The above results clearly elucidate the critical role of dimers in affecting the NIR-II emission from 4F (Fig. 3d). For unimolecular 4F, 4F returns to the ground state through both radiative (134 ps) and non-radiative (4.6 ps) decay pathways. In 4F aggregates, the formation of dimers not only generates new near-infrared absorption peaks around 840 nm but also induces a further redshift of the original monomer absorption peak. This assignment is supported by the spectral decomposition in Fig. 2g which shows distinct absorption peaks corresponding to monomers (~760 nm) and dimers (~840 nm). Upon photoexcitation, both monomers and dimers reach the excited state. Dimers primarily return to the ground state through ultrafast *inter*NR and, to a lesser extent, through radiative decay. Notably, these two processes, especially *inter*NR, are more competitive than that of monomers, resulting in significantly reduced emission. Based on these findings, we recognize that the reduced emission in 4F aggregates essentially arises from the predominant population of dimers with weak emission and intense *inter*NR decay features. Therefore, reducing the dimer population through a nanoengineering strategy is principally applicable to creating bright NIR-II fluorophores.

### Ultrabright NIR-II fluorophore enabled by reducing dimer population

To validate our hypothesis, the dimer population in aggregates was modulated by controlling the doping concentration of 4F in F-127 solution. The resulting 4F NP2s (4F: 0.1 mg mL^−1^) and 4F NP3s (4F: 0.01 mg mL^−1^) with lower doping concentration exhibit slightly smaller hydrodynamic diameters of 40 nm and 30 nm (Fig. S15), respectively, compared to 4F NP1s (4F: 1 mg mL^−1^). These nanoparticles maintain a zeta potential of approximately −15 mV (Fig. S19) and show negligible changes in size over 15 days of storage (Fig. S20), indicating good colloidal stability^41,42^. Moreover, 4F NPs exhibited superior photostability compared to ICG (Fig. S21). The fluorescence of long-preserved 4F NPs exhibit minimal variations in intensity and spectral lineshape (Fig. S22), further confirming their outstanding optical stability. These results collectively indicate the excellent stability of 4F NPs.

**Fig. 4:**
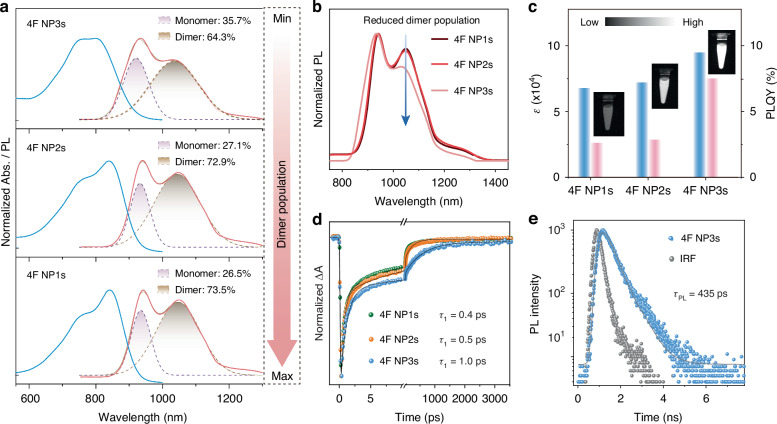
Significant alleviation of emission quenching by reducing dimer population. **a** Decomposition of absorption and PL spectra after Gaussian fitting. (blue line: Abs.; red line: PL). **b** Normalized PL spectra of 4F NPs in aqueous solution with different dimer population. **c**. The molar extinction coefficient (blue) and Φ_PL_ (red) of 4F NPs. Inset: NIR-II fluorescence images of 4F NPs. **d** Kinetics traces of 4F NPs at the representative wavelength. **e** Femtosecond transient fluorescence spectrum of 4F NP3s

4F NP2s displayed an absorption spectrum similar to that of 4F NP1s, with distinct monomer and dimer peaks at 760 nm and 840 nm (Fig. 4a), respectively. In contrast, 4F NP3s exhibited a noticeably decreased dimer-associated absorption around 840 nm and emission around 1050 nm (Fig. 4a, b). Spectral decomposition revealed a reduced dimer population from 73.5% to 64.3% in 4F NP3s (Fig. 4a), consistent with molecular dynamics simulations that showed a reduced proportion of dimers as the decrease in 4F concentration (Fig. S23).

As expected, 4F NP2s and 4F NP3s exhibited enhanced Φ_PL_, increasing from 2.6% for 4F NP1s to 2.9% and 7.5%, respectively (Fig. 4c, Fig S11, Table S3). Moreover, ε was also progressively enhanced from 6.8 × 10^4^ to 7.2 × 10^4^ and 9.5 × 10^4^ M^−1^cm^−1^ at 808 nm as aggregation decreased (Fig. S9), with 4F NP3s exhibiting an ultrahigh ε relative to unimolecular 4F (ε_808nm_ = 6.4 × 10^4^ M^−1^cm^−1^). Collectively, 4F NP3s demonstrated superior fluorescence brightness of 7.1 × 10^3^ M^−1^cm^−1^ (Fig. 4c), which nearly maintains the brightness of unimolecular 4F in THF (10.9 × 10^3^ M^−1^cm^−1^) and is 4-times higher than that of 4F NP1s (1.8 × 10^3^ M^−1^cm^−1^). Notably, the brightness of 4F NP3s is also ~5-times greater than that of ICG (1560 M^−1^ cm^−1^), repressing the top-level NIR-II fluorescence brightness. These phenomena suggest the successful regulation of dimer population in alleviating ACQ, thereby improving the NIR-II brightness of 4F aggregates.

Furthermore, ultrafast spectroscopy analysis provided an in-depth understanding of the elevated Φ_PL_ of 4F NP3s_._ The representative kinetic curves within the GSB region indicate a decelerated *inter*NR decay in 4F NP2s and 4F NP3s, reflected by extended time constants from 0.49 ps to 0.98 ps (Fig. 4d, Table S5). Concurrently, the dimer emission exhibited an extended lifetime, increasing from 9.4 ps to 11.6 ps. This inefficient *inter*NR and extended fluorescence lifetime from the dimer can be attributed to the increased intermolecular distance within the dimer (Table S4), which creates significant space for monomer fluorescence. Femtosecond transient fluorescence spectroscopy measured the fluorescence lifetime of 4F NP3s as 435 ps (Fig. 4e), which aligns well with the long-lived component (496 ps) extracted by the fs-TA within GSB regions. Thus, the time constants of 227 ps for 4F NP2s, 179 ps for 4F NP1s, and 134 ps for 4F were reasonably attributed to monomer fluorescence. Moreover, the populations of this monomer fluorescence increased from 9.6% to 20.2% as the dimer populations decreased (Table S5), supporting the elevated Φ_PL_ of 4F NP3s. Unfortunately, our femtosecond transient fluorescence spectroscopy could not measure the fluorescence lifetimes for 4F NP2s, 4F NP1s, and 4F due to its detection limit (~300 ps). Together, these results reinforce the critical role of dimers in modulating NIR-II brightness from 4F aggregates. Dimers with extended intermolecular distances not only preserve the intense long-wavelength absorption but also alleviate the long-standing ACQ issue, offering valuable insights for designing brighter fluorophores beyond NIR-II fluorescence imaging applications.

### High-performance NIR-II fluorescence imaging in vivo

**Fig. 5:**
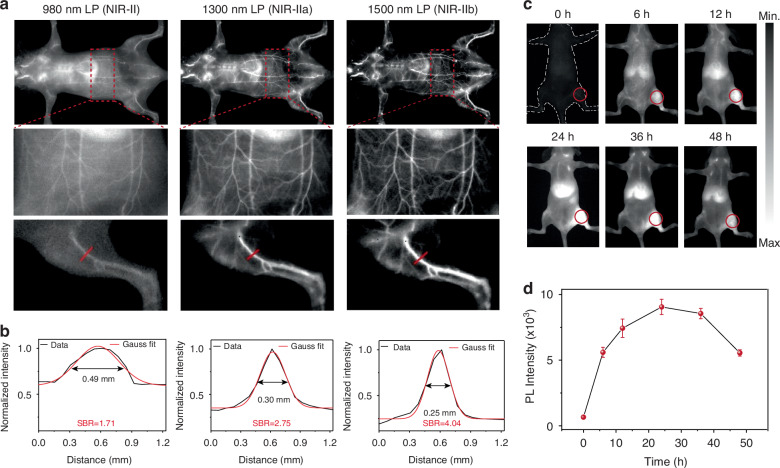
High-performance NIR-II fluorescence imaging in vivo. **a** NIR-II fluorescence imaging of blood vessels in living mice with different filters, 5 minutes post-injection of 4F NP3s (200 μL, 1 mg mL^−1^). **b** The cross-sectional fluorescence intensity profile fitted with Gauss along red lines. **c** NIR-II fluorescence images at different times after intravenous injection with 4F NP3s (200 μL, 1 mg mL^−1^) under 808 nm excitation. **d** Time-dependent NIR-II fluorescence intensity within osteosarcoma region

The exceptional brightness of 4F NP3s demonstrates significant potential for in vivo NIR-II fluorescence imaging^43,44^. Prior to in vivo experiments, the toxicity of 4F NP3s was first examined in MC3T3-E1 cell. 4F NP3s exhibit negligible cytotoxicity even at high concentrations up to 100 μg mL^−1^, indicating excellent in vitro biocompatibility (Fig. S24). The NIR-II fluorescence imaging performance of 4F NP3s was evaluated and compared with that of the clinically approved contrast agent ICG. A solution of either 4F NP3s or ICG (200 μL, 1 mg mL^−1^) was intravenously injected into caudal veins of mice. The whole-body fluorescence angiography was performed using an InGaAs detector equipped with different long wavelength pass filters (1000, 1300, and 1500 nm) (Fig. 5a). The imaging results revealed that the spatial resolution of 4F NP3s notably outperformed that of ICG (Fig. S25). Impressively, in the NIR-IIb window (1500 nm LP), 4F NP3s provided clear visualization of blood vessels with significantly reduced background noise, offering remarkable contrast compared to the less distinct vessels observed in the conventional NIR-II region (980 and 1300 nm LP filters). High-magnification imaging of hind-limb vessels provided detailed anatomical information, with full width at half-maxima (FWHM) measurements of 0.49, 0.30, and 0.25 mm for 980, 1300, and 1500 nm LP, respectively, demonstrating the superior resolution afforded by NIR-IIb imaging (Fig. 5b). Meanwhile, the NIR-IIb region exhibited the highest signal-to-background ratio (SBR) of 4.04, surpassing the SBRs of 1.71 and 2.75 for 980 and 1300 nm LP, respectively. Importantly, small vessels branching around the abdominal vessels were clearly distinguished in the NIR-IIb region, whereas no clear image could be obtained in the conventional NIR-II window (Fig. 5a). These findings demonstrate high-performance fluorescence imaging in vivo with superior SBR using 4F NP3s.

4F NP3s further demonstrate its potential in deep-seated tumor imaging using an osteosarcoma-bearing BALB/c nude mouse mode^45^. As shown in Fig. 5c, distinct NIR-II fluorescence signals were observed at the tumor sites after 6 h post-administration, with signal intensity progressively increasing and peaking at 24 hours. These results indicate that 4F NP3s can achieve deep penetration imaging and effectively accumulate in the tumor region through the enhanced permeability and retention (EPR) effect. Even after 36 hours post-injection, the fluorescence signals at the tumor sites diminished but maintained satisfactory brightness, highlighting the potential of 4F NP3s as a long-term NIR-II fluorescence imaging probe for deep-seated tumor navigation (Fig. 5d).

To assess the biodistribution and biocompatibility of 4F NP3s, the osteosarcoma and major organs were collected 48 hours post-injection. NIR-II imaging revealed that the nanoparticles primarily accumulated in the liver and spleen, suggesting a metabolic pathway via the hepatobiliary system (Fig. S26). Furthermore, hematoxylin–eosin (H&E) staining of the major organs showed no significant hydropic damage or necrotic lesions, indicating the good biosafety of 4F NP3s (Fig. S27). Overall, 4F NP3s demonstrate high-performance NIR-IIb angiography with sharp resolution and excellent potential as tumor navigation contrasts for phototheranostic applications.

## Discussion

This study presents a multidisciplinary approach to significantly alleviate ACQ of 4F by elucidating the exact role of dimer in enhancing NIR-II fluorescence imaging. Our findings reveal the coexistence of multiple dimer architectures alongside monomers of 4F within its aggregate, with dimer population predominating. Notably, dimers exhibit significantly weaker emission and more intense *inter*NR compared to monomers. Together, the predominant population of dimers, characterized by weak emission and intense *inter*NR decay, underlies the ACQ observed in 4F aggregates. Building on this finding, reducing the dimer population leads to the development of ultrabright 4F NP3s, which enable high-resolution imaging of vascular blood vessels and deep-seated osteosarcoma.

The ring-fused 4F offers multiple advantages for designing novel NIR-II fluorophores. The 4F molecule features an intramolecular noncovalent S···O interaction that promotes a giant π-conjugation and a rigid molecular structure. The extensive π-conjugation facilitates both intense absorption and high Φ_PL_, while the rigid structure minimizes nonradiative energy loss by molecular motions such as conformational dynamics. Together, these features contribute significantly to the high NIR-II Φ_PL_ for 4F. This noncovalent conformational lock addresses the challenges associated with traditional D-A type NIR-II fluorophores, which often possess twisted structures that result in weaker absorption at longer wavelengths.

These robust properties afford great potential for the clinical translation of 4F, although clinical and translational path is undeniably complex. For effective clinical imaging, fluorophores must exhibit high fluorescence brightness and photostability, which are all satisfied by 4F NP3s. The ultrahigh NIR-II brightness (7125 M^−1^ cm^−1^) represents one of the highest levels of brightness among available NIR-II fluorophores (Table S1, 2), offering unprecedented potential in high-resolution visualization of vital structures such as blood vessels and malignant lesions (Fig. 5a, b). Moreover, their long-term colloidal and optical stability facilitates real-time and dynamic high-resolution imaging (Fig. 5b, Fig. S19–22), which could revolutionize surgical procedures and therapeutic interventions. Secondly, fluorophores are non-toxic and can be safely metabolized or excreted by the body is paramount. Biosafety analyses of 4 F NP3s indicate negligible long-term toxicity, with metabolism primarily via liver (Fig. S26, 27).

Beyond great clinical potential in fluorescence imaging, this study also proposes and validates that the predominant population of dimers, characterized by weak emission and intense *inter*NR decay, underlies the ACQ observed in 4F aggregates. This marks a significant advancement in addressing the longstanding challenges of ACQ, a common phenomenon in various applications that rely on strong and stable emissions. The success of this approach introduces an innovative strategy for the development of ultrabright organic π-conjugated materials even in aggregated states that is unattainable in traditional strategy.

In conclusion, this study not only offers novel insights into overcoming the long-standing challenge of ACQ but also paves the way for creating highly efficient and stable NIR-II fluorophores, with potential applications extending beyond NIR-II fluorescence imaging. Therefore, we believe this work will attract substantial interest from a broad readership across biomedical science, photonics, material science, chemistry, optoelectronics, and related disciplines.

## Materials and methods

### Materials

All the chemicals and solvents were purchased from Energy-Chemical, Sigma-Aldrich and J*&*K without additional treatment before use. 4,4,9,9-tetrakis (4-hexylphenyl) -2,7-bis (trimethylstannyl) −4,9-dihydro-s-indaceno [1,2-b: 5,6-b] dithiophene, 5-bromo-4-((2-ethylhexyl) oxy) thiophene-2-carbaldehyde, 2-(5,6-difluoro-3-oxo-2,3-dihydro-1H-inden-1-ylidene)malononitrile was purchased from Nanjing Zhiyan Technology Co., Ltd. Tris (dibenzylideneacetone) dipalladium, tri (o-tolyl) phosphine, were purchased from J&K Scientific Ltd. Pluronic F-127 were purchased from Sigma-Aldrich.

### Synthesis of 4F

The synthesis methods for all compounds and their characterization data are detailed in the Supplementary Information, Scheme S1, Fig. S3–8.

### General preparation of nanoparticles

4F (1.0 mg) in 1 mL THF was swiftly dropped into the Pluronic F-127 aqueous solution (10.0 mg in 10 mL H_2_O) under sonication. THF was then removed by argon blowing over the solution surface under stirring at 25 °C. A blue aqueous solution was then obtained as 4F NP1s. Similarly, 4F NP2s and 4F NP3 were constructed with different concentrations of 4F as 0.1 mg mL^−1^ and 0.01 mg mL^−1^, respectively. The aqueous solution was concentrated with a centrifugal filter and washed several times. The resultant products were concentrated and filtered through a 0.45 μm filter for future experiments.

### NIR-II photoluminescence quantum yield

IR-26 with a NIR-II Φ_PL_ of 0.5% was chosen as a standard. A stock solution of IR-26 in 1,2-dichloroethane was diluted to obtain a series of samples with absorbance values of ~0.10, ~0.08, ~0.06, ~0.04, and ~0.02 at 808 nm, respectively. The NIR-II emission spectra of the five solutions were collected with an 850 nm LP filter to cut off the excitation light (808 nm). Then the emission spectra were integrated in the wavelength region of 900–1500 nm. The obtained emission integration was plotted against absorbance intensity to establish a linear correlation. The same procedures were applied to 4F in THF and 4F NPs in water too. The Φ_PL_ was calculated using the following formula:

$${\Phi }_{{\rm{sample}}}={\Phi }_{{\rm{ref}}}\times \frac{{{slope}}_{{sample}}}{{{slope}}_{{ref}}}\times {\left(\frac{{\eta }_{{sample}}}{{\eta }_{{ref}}}\right)}^{2}$$

where Φ_ref_ is 0.5% and η_sample_ represent the refractive index of the corresponding solvent, mainly including THF and water. η_ref_ represent the refractive index of 1,2-dichloroethane.

### Ultrafast spectroscopy

The fs-TA spectra were obtained using an amplified Ti:sapphire laser system that produces a fundamental 800 nm output with a pulse width of 120 fs and a 1 kHz repetition rate (Solstice Ace, Spectra-Physics). This laser system provides an average power of 7 W, divided into two beams in a 7:3 ratio for pump and probe, respectively. The first high-power energy beam is directed into an optical parametric amplifier (TOPAS-prime, Light Conversion) to produce tunable pump wavelengths ranging from 245 nm to 2600 nm. The second beam was focused onto a 3 mm Sapphire plate and filtered with an 800 nm short-pass filter to remove the fundamental 800 nm laser pulse, creating a white light continuum (WLC) used as the probe pulse between 420 and 850 nm. A high-precision computer-controlled translation stage-managed the pump-probe delay, achieving a 14 fs precision within an 8 ns time window. The relative polarizations between the pump and probe beams were aligned to the magic angle (54.7°) to avoid rotational depolarization effects. The fs-TA spectra measurements were conducted in a quartz cuvette with a 2 mm path length. Before passing through the sample, the probe beam is split into signal and reference beams. The signal beam traversed the sample, and the reference beam directly entered fiber-coupled spectrographs, monitoring probe beam intensity fluctuations. Pump-induced transmission changes (ΔOD) were created by alternating probe pulses with a chopper wheel on the pump beam and monitored with fiber-coupled spectrographs equipped with linear array detectors. A multi-exponential function was employed to fit the kinetic curves.

Femtosecond transient fluorescence spectra were measured on a fluorophotometer assembled in our laboratory, in which FPGA based Time-to-digital converter (TDC) module modified TCSPC (FF4, Orient KOJI Ltd., CHN) was inserted between the Spectra-Physics Tsunami Oscillator (80 MHz, 800 nm) laser and the detector of a commercial Jobin Yvon Horiba FluoroMax-4. The NIR-II fluorescence lifetime was acquired with a NIR-II PMT unit (H10330C, Hamamatsu).

### Animal handling

All the animal studies were performed following the Guide for the Institutional Animal Care and Use Committee and the protocol approved by the Animal Health and Use Committee of Northwestern Polytechnical University. Balb/c mice were purchased from Jiangsu KeyGEN Biotechnology Co., Ltd. for in vivo angiography. Female BALB/c nude mice were used to develop the osteosarcoma mice model. 143B cells (3.0 × 10^7^ mL^−1^, 30.0 μL in PBS) were subcutaneously injected into the bone marrow cavity of the left tibia. Tumors were allowed to grow for approximately 18 days to reach a volume of about 80–100 mm^3^.

### Whole-body vascular imaging

Healthy Balb/c mice were used for in vivo vascular imaging. Hair from the limbs, chest, and abdominal region of the mic was removed using depilatory gel. Then, the mice were administered with 4F NP3s (1 mg mL^−1^, 200 μL) via tail vein injection. In vivo NIR-II fluorescence imaging was performed using a NIR-II imaging system (Wuhan Grand-imaging Technology Co., Ltd) under 808 nm photoexcitation, equipped with LP 980 nm, LP 1300 nm, LP 1500 nm filters.

### NIR-II Imaging of osteosarcoma mice

4 F NP3s (1 mg mL^−1^, 200 μL) were intravenously injected into the mice through the tail vein. NIR-II imaging was performed upon excitation at 808 nm in NIR-II fluorescence imaging system with 980 nm LP filter. The fluorescence images were captured at 0, 6, 12, 24, 36, and 48 h post-injection.

## Supplementary information

Supplementary Information

## Data Availability

The authors declare that the data supporting the findings of this study are provided in the Supporting Information file. All data are available from the authors upon request.
