# Supplementary material for: Alleviating NIR-II emission quenching in ring-fused fluorophore via manipulating dimer populations for superior fluorescence imaging
Source: Light Sci Appl. 2025 Mar 4;14:109. doi: 10.1038/s41377-025-01787-0 (PMC11880303; doi:10.1038/s41377-025-01787-0)
Supplement: Supplementary file 1 — Supplementary Information [file 41377_2025_1787_MOESM1_ESM.pdf]

Supporting Information

Alleviating NIR-II emission quenching in ring-fused fluorophore via manipulating dimer populations for superior fluorescence imaging

Xiaofei Miao[1,2], Mingxuan Jia[2], Xianwei Weng[1], Jie Zhang[1], Yonghui Pan[1], Hui Zhao[1],

Zhongzheng Yu[3], Quli Fan[1] and Wenbo Hu[1,2]**

[1] State Key Laboratory of Flexible Electronics (LoFE) & Institute of Advanced Materials (IAM), Nanjing University of Posts & Telecommunications, Nanjing, 210023, China.

Email: iamqlfan@njupt.edu.cn

[2] State Key Laboratory of Flexible Electronics (LoFE) & Institute of Flexible Electronics (IFE), Northwestern Polytechnical University, Xi'an, 710072, China.

E-mail: iamwbhu@nwpu.edu.cn

[3] Cavendish Laboratory, University of Cambridge, Cambridge CB3 0HE, United Kingdom.

Instruments and characteristic:

Nuclear magnetic resonance (NMR) spectra were measured by using a Bruker Ultra Shield Plus 400 MHz. Mass spectra were obtained on a matrix-assisted laser desorption/ionization time of flight mass spectrometry MS (MALDI-TOF, Bruker AutoFlex III system). Dynamic light scattering (DLS) was performed on a particle size analyzer (NanoBrook 90Plus, Brookhaven Instruments Corporation). Transmission electron microscopy (TEM) images were acquired from a HT7700 transmission electron microscope. The steady-state near-infrared absorption spectra were collected on a SHIMADZU UV-3600 PLUS ultraviolet-visible-near-infrared (UV-Vis-NIR) spectrophotometer. The NIR-II fluorescence spectra were acquired on a Fluorolog 3 spectrophotometer (Horiba) equipped with an 808 nm diode laser and an InGaAs NIR detector. fs-TA spectrum was performed by our home-built system, as described by in our previous work[1,2]. Femtosecond Transient fluorescence spectra were measured on a fluorophotometer assembled in our laboratory, in which Time-to-digital converter (TDC) module modified TCSPC (FF4, Orient KOJI Ltd., CHN) was inserted between the Spectra-Physics Tsunami Oscillator (80 MHz, 800 nm) laser and the detector of a commercial Jobin Yvon Horiba FluoroMax-4. The NIR-II fluorescence lifetime was detected with an NIR-II PMT unit (H10330C, Hamamatsu). NIR-II fluorescence imaging *in vivo* was conducted on an NIR-II imaging system (Wuhan Grandimaging Technology Co., Ltd).

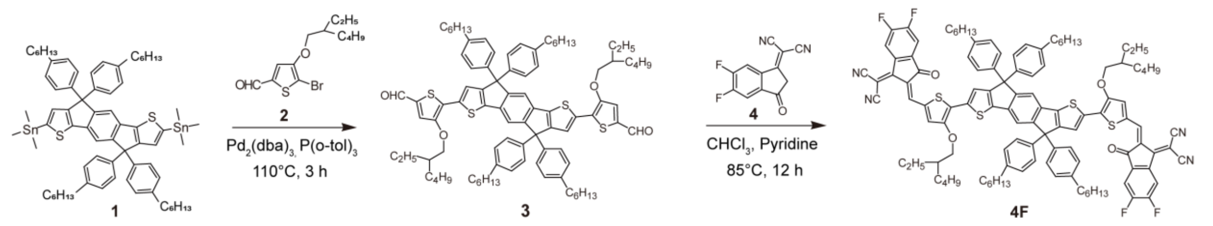

Scheme S1. The synthetic route of 4F.

Synthesis of compound 3:

Compound 3 was synthesized via a typical Still coupling. Compound 1 (2.50 g, 2 mmol) and compound 2 (1.91 g, 6 mmol) were dissolved in 10 mL of toluene. The system was then degassed and filled with argon for 30 min. The catalysts Pd_2 $(dba)_3$ and tri(o-tolyl) phosphine were added, and the reaction mixture was refluxed in the dark at 110°C for 3 hours. After cooling into room temperature, the mixture was transferred to a separatory funnel and extracted with dichloromethane (DCM) three times. The combined organic phase was dried over anhydrous $MgSO_4$. The solvent was concentrated using a rotary evaporator, and the residue was further purified by column chromatography on silica using petroleum ether: DCM (1:1) as the eluent. The final product, compound 3, was obtained as an orange oil. (2.22 g, yield: 80%). ^{1}H NMR (400 MHz, $CDCl_3$) δ 9.76 (s, 2H), 7.66 (dd, J = 6.4, 3.1 Hz, 2H), 7.46 (dd, J = 8.7, 2.9 Hz, 8H), 7.41 (s, 2H), 7.21 (d, J = 8.2 Hz, 8H), 7.12 (s, 2H), 4.09 (d, J = 5.4 Hz, 4H), 2.63-2.56 (m, 8H), 1.83 (dt, J = 12.0, 5.8 Hz, 2H), 1.67-1.53 (m, 16H), 1.40-1.30 (m, 24H), 0.97 (t,

J = 7.5 Hz, 8H), 0.91 (dt, *J* = 10.1, 5.2 Hz, 24H). ^{13}C NMR (101 MHz, $CDCl_3$) δ 188.93, 181.59, 156.39, 153.77, 153.20, 143.35, 142.82, 141.67, 141.62, 136.69, 135.63, 135.46, 134.85, 130.52, 128.99, 128.42, 127.89, 127.42, 125.47, 123.32, 121.43, 117.52, 74.34, 62.92, 39.66, 35.60, 31.74, 31.35, 30.51, 29.17, 29.07, 23.88, 23.01, 22.61, 14.11, 11.16.

Synthesis of 4F:

3- (dicyanomethylidene)indan-1-one (1.15 g, 5 mmol) and compound 3 (2.08 g, 1.5 mmol) were dissolved in 10 mL of chloroform, with the addition of 1 mL of pyridine. The system was then degassed and filled with argon for 30 min. The mixture was refluxed in the dark at 85°C for 12 hours. After cooling to room temperature, the solution was transferred to a separatory funnel and extracted with dichloromethane (DCM) three times. The organic phase was dried over anhydrous $MgSO_4$, and the solvent was concentrated using a rotary evaporator. The residue was further purified by column chromatography on silica using petroleum ether: DCM (1:1) as the eluent. The final product, 4F, was obtained as a dark green solid. (1.6 g, yield: 60%). ^{1}H NMR (400 MHz, $CDCl_3$) δ 8.71 (s, 2H), 8.53 (dd, *J* = 9.9, 6.5 Hz, 2H), 7.69-7.61 (m, 4H), 7.50 (d, *J* = 13.1 Hz, 4H), 7.21 (d, *J* = 8.1 Hz, 8H), 7.13 (d, *J* = 8.2 Hz, 8H), 4.13 (d, *J* = 5.3 Hz, 4H), 2.67-2.53 (m, 8H), 1.93-1.83 (m, 2H), 1.67-1.55 (m, 16H), 1.34 (dt, *J* = 20.3, 8.8 Hz, 32H), 0.99 (t, *J* = 7.4 Hz, 6H), 0.91 (dt, *J* = 13.4, 6.8 Hz, 18H). ^{13}C NMR (101 MHz, $CDCl_3$) δ 186.20, 158.16, 157.85, 154.80, 154.74, 147.08, 142.00, 141.08, 137.79, 137.09, 136.37, 136.06, 131.54, 129.82, 128.58, 127.88, 123.65, 120.70, 118.08, 114.62, 114.58, 74.64, 68.49, 62.90, 53.45, 39.59, 35.60, 31.75, 31.38, 30.51, 29.15, 29.05, 23.89, 23.01, 22.62, 14.13, 11.15.

Quantum-chemical calculations:

All density functional theory (DFT) and time-dependent DFT (TD-DFT) calculations were performed with the Gaussian 16 package.[3] The ground-state and excited-state geometries of 4F monomer and dimers were fully optimized at ωB97XD/6-31G (d, p) level. All the electronic properties were calculated at the ωB97XD/6-311G (d, p) level of theory based on the optimized geometries. The electron-hole[4] and weak interaction investigation (Independent Gradient Model based on Hirshfeld partition, IGMH)[5] were carried out by Multiwfn (version 3.8 dev) program[6,7].

Molecular dynamics simulation:

All our MD simulations in this work were performed by GROMACS-2020.6 package[8]. All atom types and their structural parameters were taken from the general amber force field (GAFF)[9]. To mimic amorphous 4F aggregates, we first performed the large-scale MD simulations to obtain the various dimer conformations in aqueous solution. Our MD simulations are sufficiently long for 4F to reach stable amorphous states. We performed energy minimization, followed by 5 ns equilibrium simulations with position restrains in three dimensions for ZnP under NPT (T = 298.15 K, room temperature) ensemble with temperature annealed from 150 to 373 and cooling to 298.15 K in the first 5 ns. Finally, we perform 50 ns production MD simulations with 2 different concentrations (15 and 30 4F molecules in 9 nm^3

water environment) under the NPT (T = 298.15 K and P = 1 bar) ensemble. The cutoff distance for VDW interactions was 1.0 nm. All bond lengths were constrained via the LINCS algorithm. The aggregation results of 4F dimers are illustrated by the representative snapshots extracted from MD trajectories for systems at different concentrations.

Cytotoxicity assay. MC3T3-E1 cells were cultured with fresh medium (90% DMEM + 10% FBS) within an incubator set to 5% CO_2 and 37°C. Cells, seeded in 96-well plates, were treated with different concentrations (0, 10, 20, 40, 80 and 100 μg mL^{-1}) of 4F NP3s for 24 h. After that, the medium was then removed, 10%·Cell Counting·Kit-8 (CCK-8) solution was added to each well and incubated for an additional 30·min·at 37°C. Then, the absorbance at 450 nm·was measured using a microplate reader.

Table S1 Photophysical characterization of the state-of-the-art and previously reported NIR-II fluorophores.

Fluorophore	$\lambda_{abs.}$ (nm)	$\varepsilon \times 10^3$ [M^{-1} cm^{-1}]	λ_{em} (nm)	Φ_f (%)	Brightness [M^{-1} cm^{-1}]	Ref.
4F NP3s 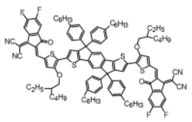	840	95	1040	7.5	7125	This work
2PhNVDPP NPs 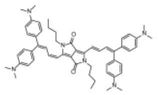	703	18	929/1013	0.15	27	*J. Am. Chem. Soc.* **146**, 32582−32594 (2024)[10]
4TT-PPT NPs 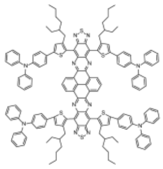	700	9.8	~1000	1.94	190.3	*Angew. Chem. Int. Ed*, e202417865 (2024) [11]
TEEITQ NPs 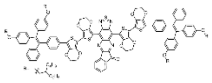	855	16.8	1102	0.26	43.7	*Angew. Chem. Int. Ed.*, e202413219 (2024) [12]
BNDI-Me NPs 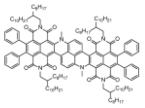	859	164	1104	1.40	2296.0	*Adv. Sci.* **10**, e2204695 (2023) [13]
IR-FFCHP NPs 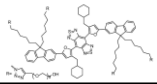	~760	~12.5	1038	7.30	912.5	*Research* **6**, 0039 (2023) [14]
TT3-oCB 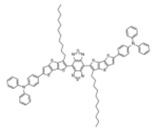	784	20.2	1062	4.6	929.2	*J. Am. Chem. Soc.* **144**, 15391-15402 (2022) [15]
TTQiT NPs 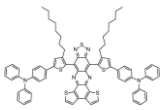	755	38.9	1102	3.7	1439.3	*Small* **18**, e2105362 (2022) [16]

THPP 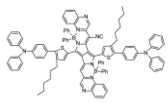	970	240	990	6	14400	*Angew. Chem. Int. Ed.* **60**, 3967-3973 (2021) [17]
CPTIC NFs 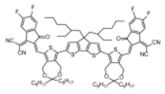	876	145	1110	3.9	565.5	*Nano Res.* **13**, 2570-2575 (2020) [18]
BTC1070 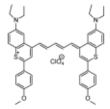	1015	45	1065	0.16	72	*Nat. Commun.* **10**, 1058 (2019) [19]
IR-BGMC6 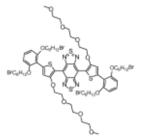	737	2.4	1036	1.5	36	*Chem. Sci.* **10**, 326-332 (2018) [20]
IR-FTAP 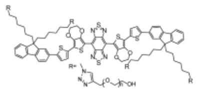	733	5	1048	5.3	263	*J. Am. Chem. Soc.* **140**, 1715-1724 (2018) [21]
IR-BGP11 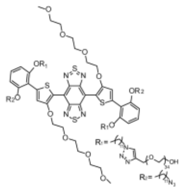	763	10.8	1047	3	324	*Adv. Funct. Mater.* **28**, 1804956 (2018) [22]
IR-FTP 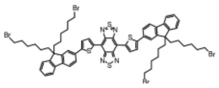	828	6.9	1047	0.02	1.4	*Adv. Mater.* **29**, 1605497 (2017) [23]

Table S2 Photophysical characterization of the reported ring-fused NIR-II fluorophores in recent five years.

Fluorophore	$\lambda_{abs.}$ (nm)	$\varepsilon \times 10^3$ [M^{-1} cm^{-1}]	λ_{em} (nm)	Φ_f (%)	Brightness [M^{-1} cm^{-1}]	Ref.
4F NP3s 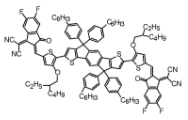	840	95	1040	7.5	7125	This work

Y6CT-NPs 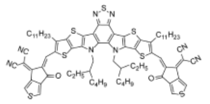	798	82.6	947/1030	8.62	7120.1	*Nat. Commun.* **15**, 5832 (2024) [24]
AS2[1:4] 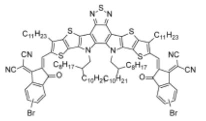	811	89.5	925/1035	1.7	1521.5	*Adv. Healthcare Mater.* **13**, 2400962 (2024) [25]
*d*BTIC-D NPs 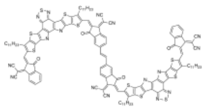	~770	140	~930/1030	2	2800	*Aggregate* **5,** e528 (2024) [26]
TPC NPs 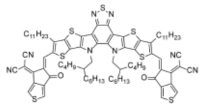	773	ND	~930/1030	9.8	ND	*Adv. Funct. Mater.* **34**, 2406483 (2024) [27]
BMIC-BO-4F NPs 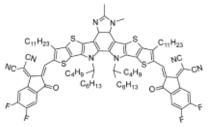	852	88	1024	2.33	2050.4	*Angew. Chem. Int. Ed.* **62**, e202303476 (2023) [28]
BTIC-δOH-2Cl NPs 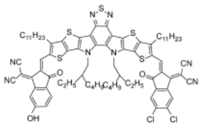	790	65.5	~1050	2.27	148.7	*Adv. Sci.* **11**, 2307569 (2023) [29]
NIR-II-H NPs 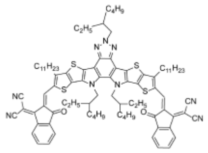	~780	ND	~900	6.6	ND	*Nano Today* **45**, 101550 (2022) [30]
BDTR9-C8 NPs 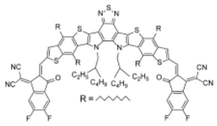	766	94.3	1054	1.89	1782.3	*Angew. Chem. Int. Ed.* **61**, e202117433 (2022) [31]

FY6-NPs 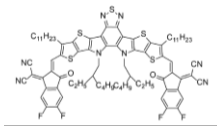	822	76.4	947/1052	4.2	3208.8	*Adv. Mater.* **35**, 2208229 (2022) [32]
IDSe-IC2F NPs 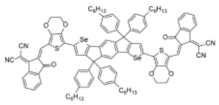	~800	ND	1036	2.1	ND	*Adv. Mater.* **34**, 2201263 (2022) [33]
COi6-4Cl NPs 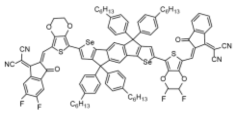	856	84	1035	5.2	4368	*Adv. Mater.* **32**, 2003471 (2022) [34]

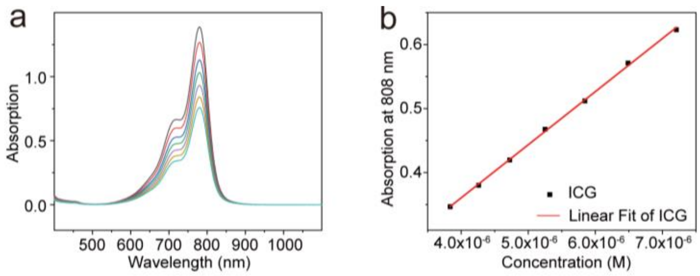

Fig. S1. The molar extinction coefficient of ICG. (a) Concentration dependence of UV-Vis-NIR absorption. (b). The plot of optical density at 808 nm versus concentration. The straight line is a linear fit to the data to get molar extinction coefficient. The molar extinction coefficient of ICG is 1560 M^{-1} cm^{-1}.

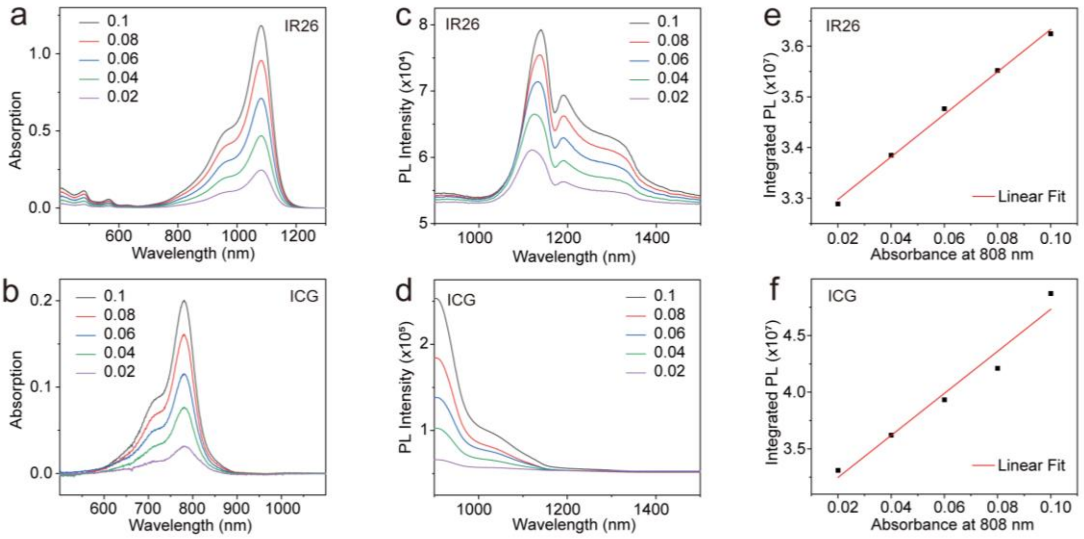

Fig. S2. NIR-II PL quantum yield of ICG. UV-Vis-NIR spectra of IR-26 (a) and ICG (b). PL spectra of IR-26 (c) and ICG (d). Integrated PL spectra of IR-26 (e) and ICG (f) as a function

of absorbance at 808 nm. The NIR-II PL quantum yield of ICG is 1.88%.

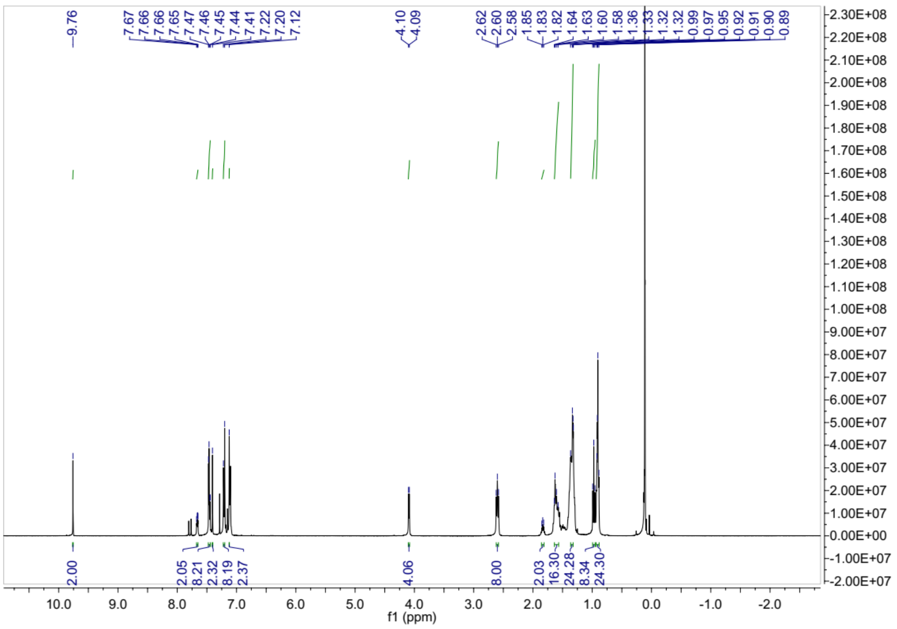

Fig. S3. ^{1}H NMR spectrum of compound 3 in $CDCl_3$.

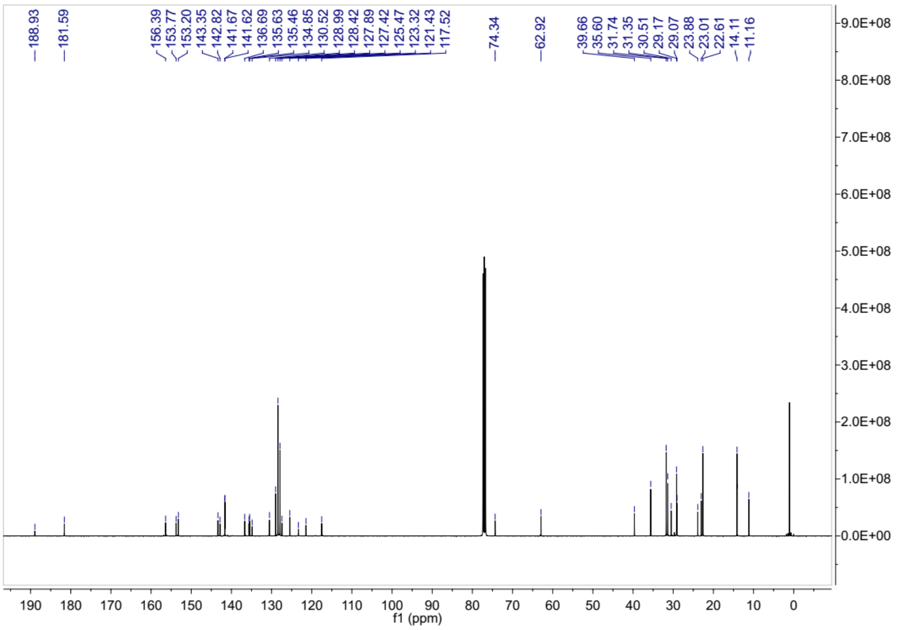

Fig. S4. ^{13}C NMR spectrum of compound 3 in $CDCl_3$.

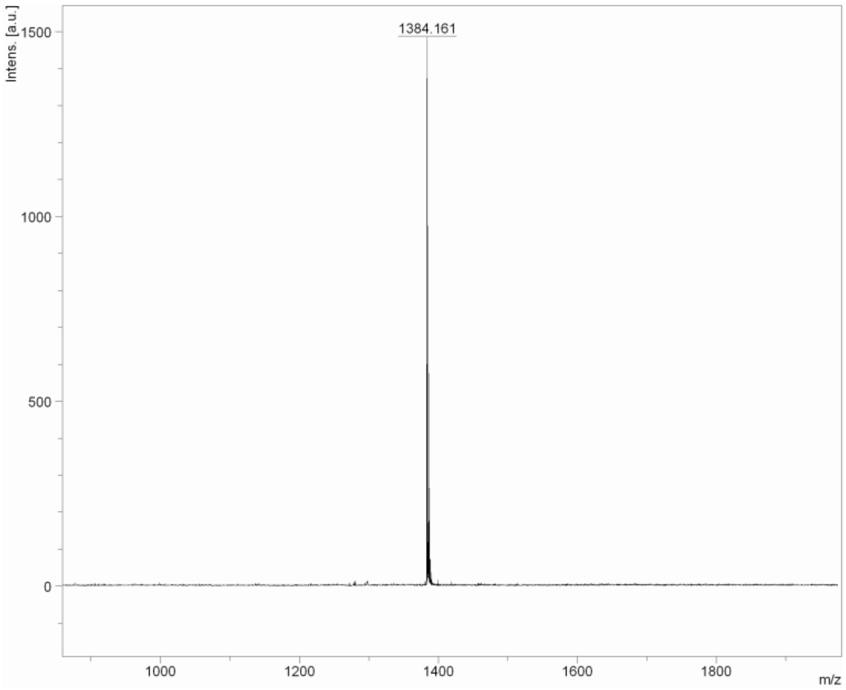

Fig. S5. MALDI-TOF of compound 3.

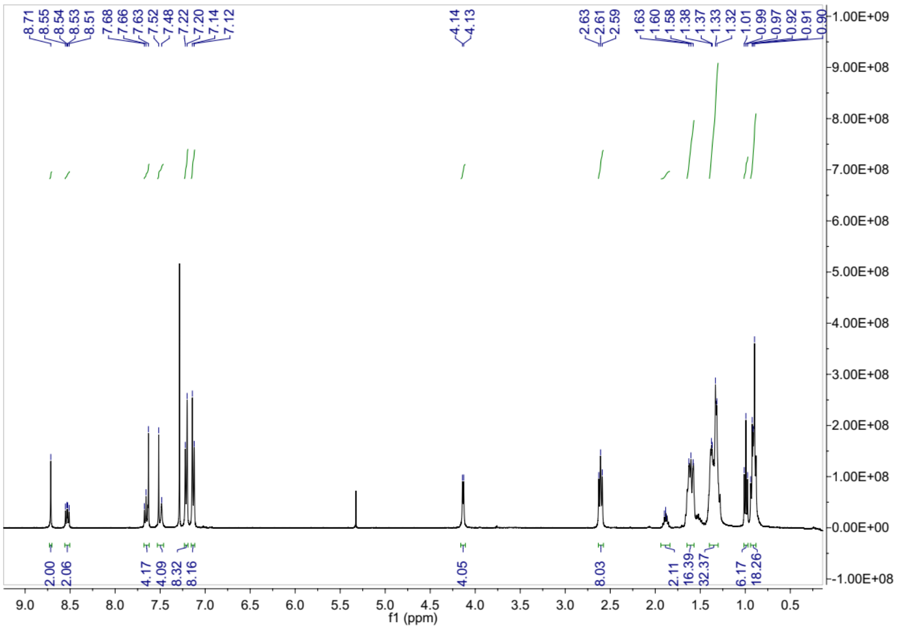

Fig. S6. ^{1}H NMR spectrum of 4F in $CDCl_3$.

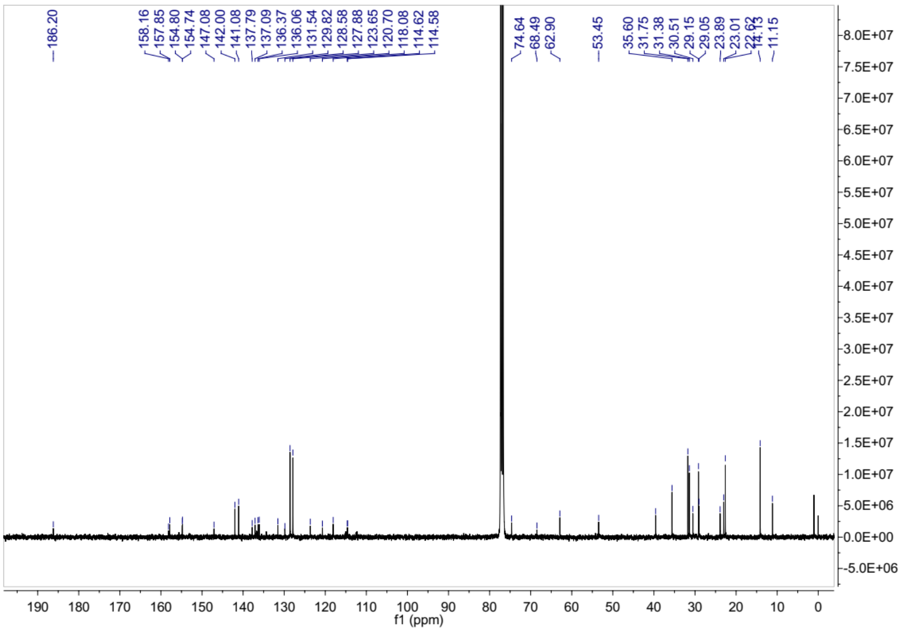

Fig. S7. ^{13}C NMR spectrum of 4F in $CDCl_3$.

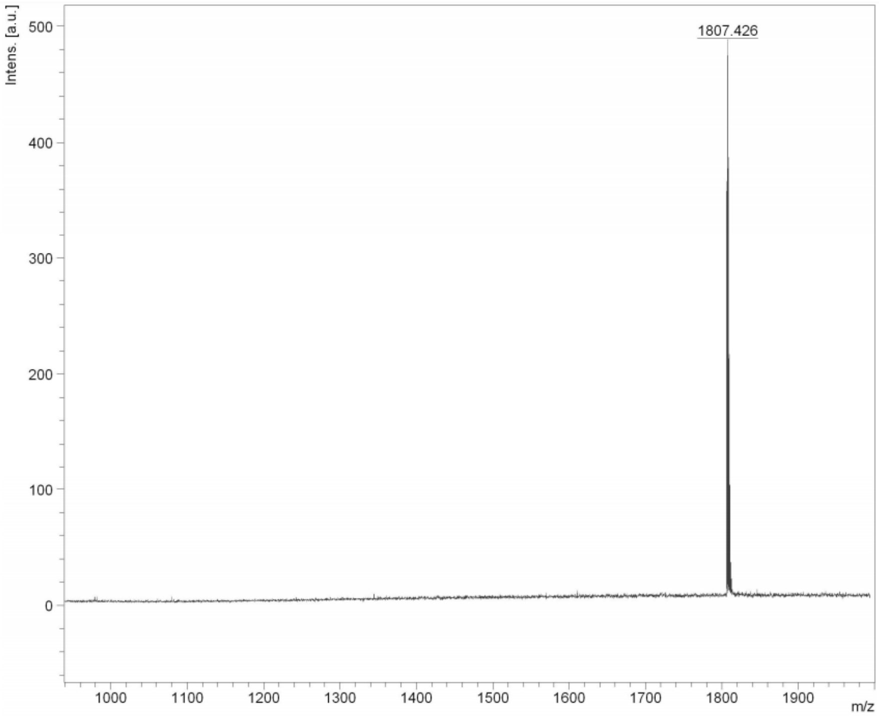

Fig. S8. MALDI-TOF of 4F.

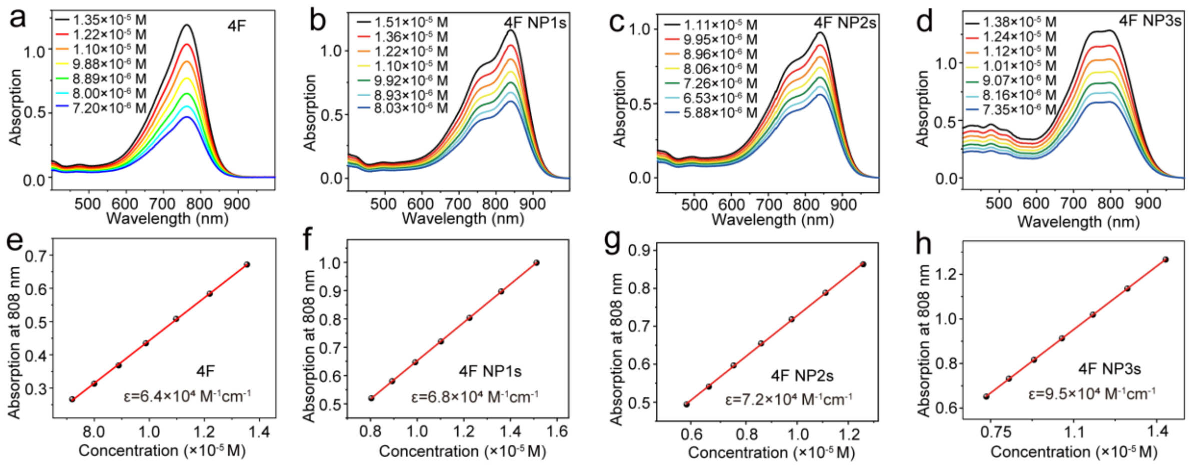

Fig. S9. The molar extinction coefficient of 4F and 4F NPs. (a-d) Concentration dependence of UV-Vis- NIR absorption. (e-h). The plot of optical density at 808 nm versus concentration. The straight line is a linear fit to the data to get molar extinction coefficient.

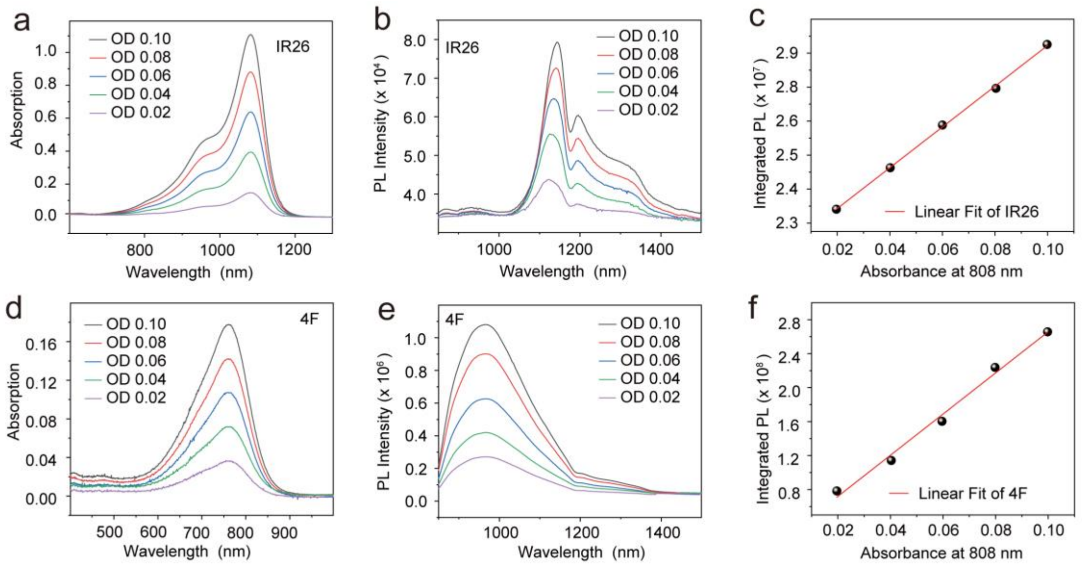

Fig. S10. NIR-II PL quantum yield of 4F. UV-Vis-NIR spectra of IR-26 (a) and 4F (d). PL spectra of IR-26 (b) and 4F (e). Integrated PL spectra of IR-26 (c) and 4F (f) as a function of 808 nm absorbance. The NIR-II PL quantum yield of 4F is 17.1%.

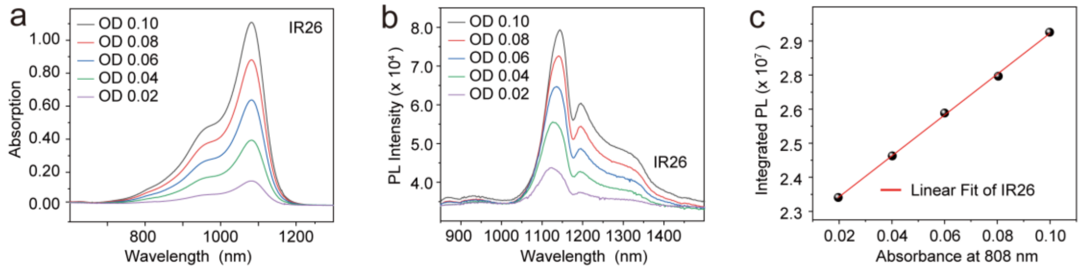

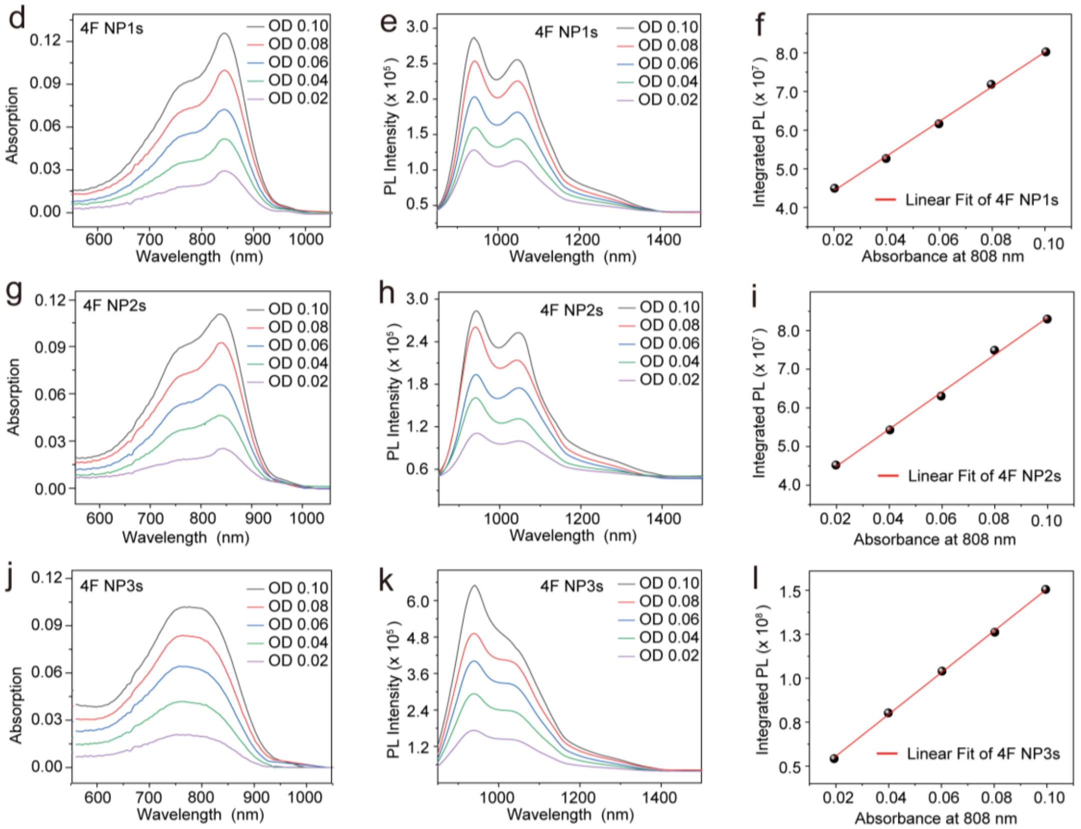

Fig. S11. NIR-II PL quantum yield of 4F NPs. UV-Vis-NIR spectra of IR-26 (a) and 4F NPs (d) (g) (j). PL spectra of IR-26 (b) and 4F NPs (e) (h) (k). Integrated PL spectra of IR-26 (c) and 4F NPs (f) (i) (l) as a function of 808 nm absorbance. The NIR- II PL quantum yield of 4F NP1s, 4F NP2s and 4F NP3s is 2.6%, 2.9% and 7.5%, respectively.

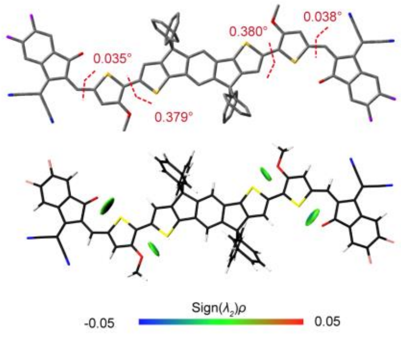

Fig. S12. Optimized molecular conformation and calculated non-covalent interactions at S_0 states of 4F. This attributes the planar architecture of 4F to an intramolecular noncovalent S···O conformational lock.

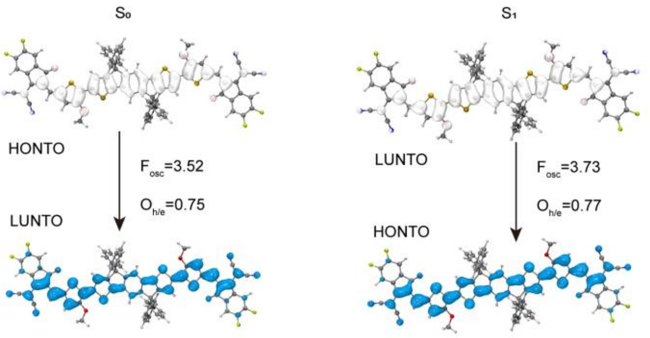

Fig. S13. NTO (hole and electron) wavefunctions for the ground state (S_0) first singlet (S_1) excited states of 4F (F_{osc}: oscillator strength; $O_{h/e}$: overlap between NTO electron and hole orbitals). This demonstrates the contributions of extensive π-conjugation to the absorption and emission of 4F, which unlike the conventional charge-transfer (CT) state typically observed in traditional D-A NIR-II fluorophores to contribute its absorption and emission.

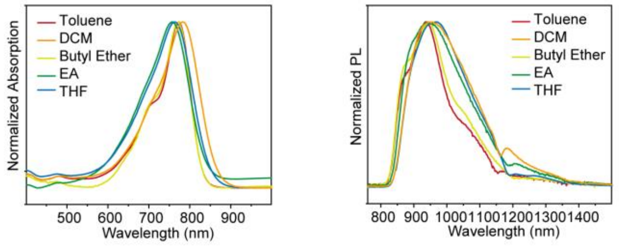

Fig. S14. Normalized absorption and PL spectra in different solvents (DCM: dichloromethane; EA: ethyl acetate; THF: tetrahydrofuran). This minimal solvation effect indicates that the intense absorption and emission of 4F primarily stem from its extensive π-conjugation, rather than from the conventional charge-transfer (CT) state typically observed in traditional D-A NIR-II fluorophores.

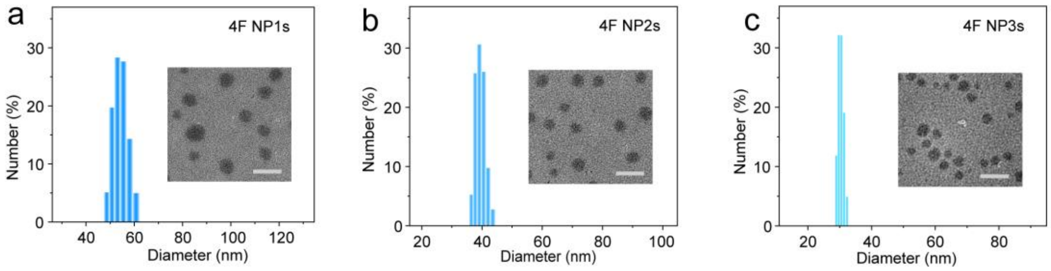

Fig. S15. The DLS analysis results and TEM images of 4F NPs (Scale bar:100 nm). The hydrodynamic diameters of 4F NP1s, 4F NP2s and 4F NP3s are about 50 nm, 40 nm and 30 nm, respectively.

Table S3. Photophysical properties of 4F in THF and 4F NPs in water.

Sample	ε 808 nm (10^4 $M^{-1}cm^{-1}$)	Φ_{PL} (%)	$\varepsilon \times \Phi_{PL}$ (10^3 $M^{-1}cm^{-1}$)
4F	6.4	17.1	10.9
4F NP1s	6.8	2.6	1.8
4F NP2s	7.2	2.9	2.1
4F NP3s	9.5	7.5	7.1

As aggregation decreased from 4F NP1s to 4F NP3s, 4F NPs exhibit enhanced molar extinction coefficient (ε) and alleviative quenching with increasing photoluminescence quantum yield (Φ_{PL}), resulting in the superior fluorescence brightness of 4F NP3s, which nearly maintains the brightness of unimolecular 4F in THF.

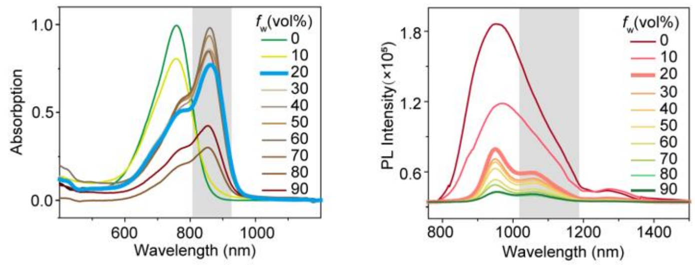

Fig. S16. The impact of aggregation on optical properties. Absorption and PL spectra of 4F in THF-H_2O solutions with varied f_{water}.

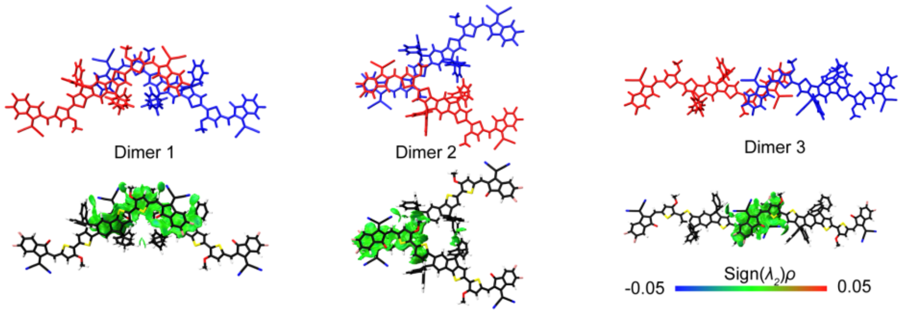

Fig. S17. Simulation of multiple dimer architectures in 4F NP1s.

Table S4. Simulation results of dimer in 4F NPs.

Sample	F_{osc}	△E (ev)	Distance (Å)
Dimer 1	1.59	1.40	3.02
Dimer 2	1.81	1.42	3.07
Dimer 3	3.85	1.38	3.26

Table S5. Fitting parameters for the representative wavelengths around 765 nm within GSB region.

Sample	τ_1 (ps)	A_1	τ_2 (ps)	A_2	τ_3 (ps)	A_3
4F	-	-	4.60	-3.9%	134	-96.1%
4F NP1s	0.43	-68.2%	7.73	-20.8%	179	-9.6%
4F NP2s	0.49	-66.0%	9.43	-21.0%	227	-11.9%
4F NP3s	0.98	-66.1%	11.6	-10.9%	496	-20.2%

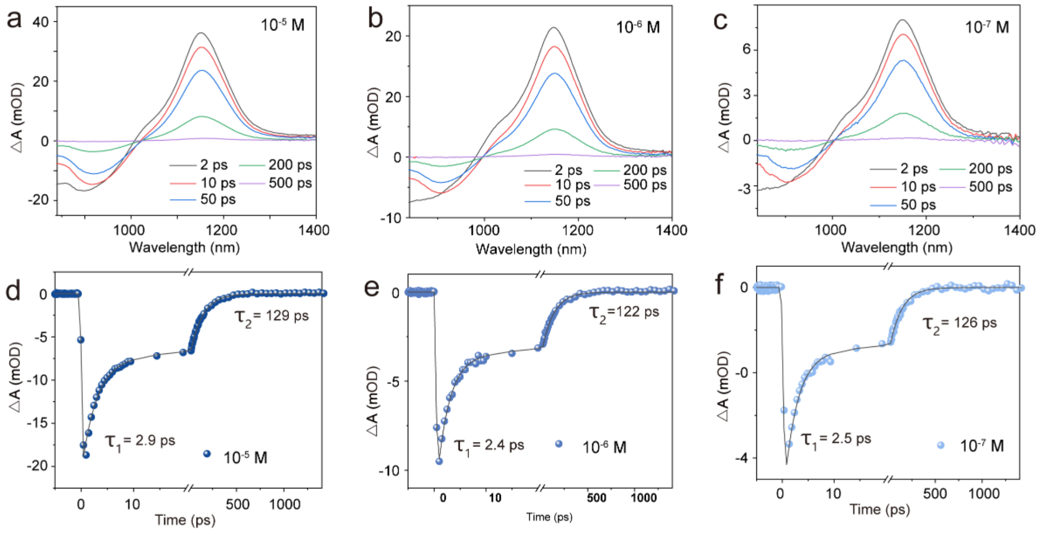

Fig. S18. (a~c) Pseudocolor fs-TA mapping of 4F in various diluted THF solutions. (d~e) Kinetics traces and fitting lines of 4F at 765 nm in various diluted THF solutions (Concentration: 10^{-5} M~10^{-7} M). In principle, a femtosecond component could arise from either *inter*NR decay or structural relaxation. In diluted solutions, 4F favors structural relaxation but disfavors *inter*NR decay. Therefore, the absence of the femtosecond component in these solutions rules out the possibility of structural relaxation, reinforcing the assignment of femtosecond component to dimer.

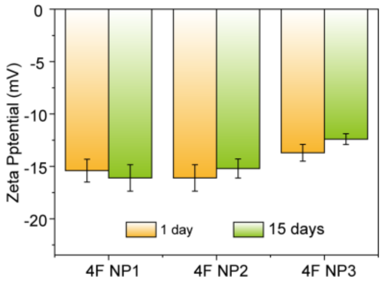

Fig. S19. Zeta potential of 4F NPs. The data are shown as mean ± SD (n = 3). These nanoparticles maintain a zeta potential of approximately -15 mV and show negligible changes over 15 days of storage, indicating good colloidal stability.

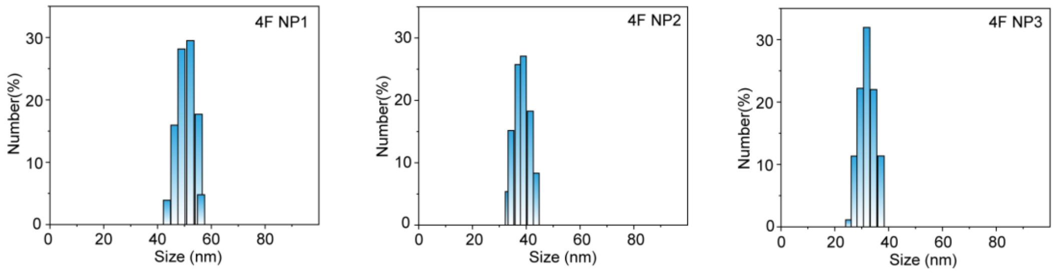

Fig. S20. The DLS analysis results of 4F NPs after 15-day storage. These nanoparticles show negligible changes in size over 15 days of storage, indicating good colloidal stability.

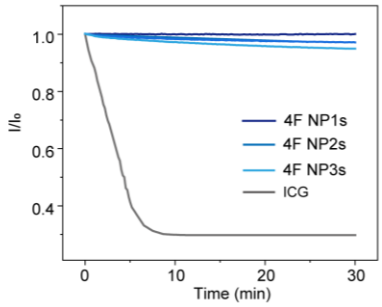

Fig. S21. Photostability 4F NPs and ICG in water upon continuous 808 nm irradiation. I represent the PL intensity of the samples at different times, while I_0 indicates the initial intensity at 0 min. 4F NPs retain over 90% of their initial fluorescence under continuous laser illumination, whereas ICG shows significant photodegradation, demonstrating their superior photostability compared to ICG.

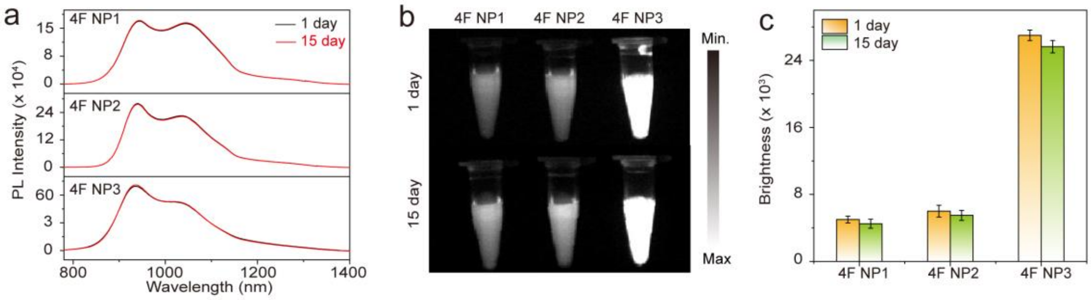

Fig. S22. (a) The PL spectra on 1- and 15-day. (b) NIR-II fluorescence images of 4F NPs on 1- and 15-day (10 μg mL^{-1}; Excitation source: 808 nm laser). (c) Quantitative fluorescence brightness of 4F NPs on 1- and 15-day (10 μg mL^{-1}; Excitation source: 808 nm laser). The fluorescence of long-preserved 4F NPs exhibit minimal variations in intensity and spectral lineshape, confirming their outstanding optical stability.

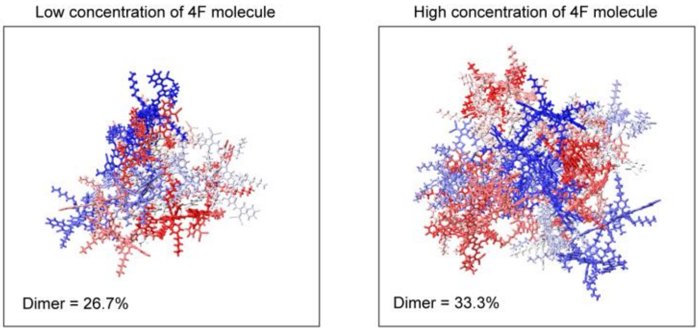

Fig. S23. Molecular dynamics simulation snapshot of 4F aggregates in H_2O. Molecular dynamics simulations show that as the 4F concentration decreases, the proportion of dimers reduces synchronously.

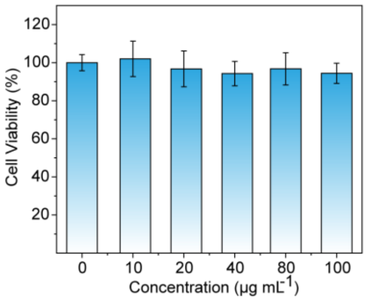

Fig. S24. 4F NP3s-concentration-dependent viability of MC3T3-E1 cells.

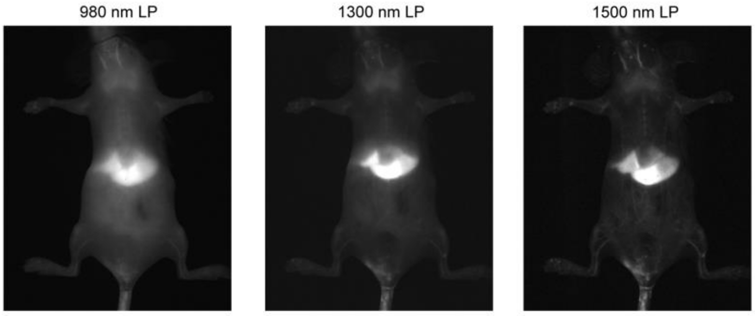

Fig. S25. NIR-II fluorescence imaging of blood vessels in living mice with different filters treated with ICG (200 μL, 1 mg mL^{-1}).

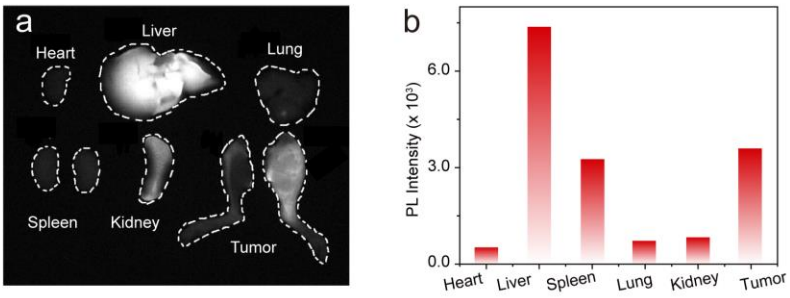

Fig. S26. (a) In vitro NIR-II fluorescence images of tumor and major organs (heart, liver, spleen,

lung, and kidney) after 48 h of intravenous injection (808 nm laser, 980 nm LP filter). (b) Quantitative NIR-II fluorescence intensity of tumor and major organs. NIR-II imaging reveals that the nanoparticles primarily accumulated in the liver and spleen, suggesting a metabolic pathway via the hepatobiliary system.

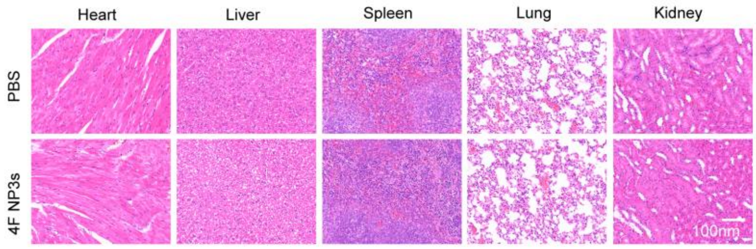

Fig. S27. Histological data (hematoxylin–eosin (H&E) images) obtained from the heart, liver, spleen, lung and kidney of different treated mice at post-injection (scale bars: 100 nm). H&E staining of the major organs shows no significant hydropic damage or necrotic lesions, indicating the good biosafety of 4F NP3s.

References

1. Miao, X. F. et al. Excimer-mediated ultrafast intermolecular nonradiative decay enables giant photothermal performance for optimized phototheranostic. *Advanced Materials* **35**, e2301739 (2023).
2. Li, M. H. et al. Tailoring the dynamics of excited-state charge transfer through conformational engineering to improve second near-infrared fluorescence for high-resolution osteosarcoma imaging. *Small Structures.* **5**, 2300483 (2024).
3. Gaussian 16, Revision A.03, M. J. Frisch, G. W. Trucks, H. B. Schlegel, G. E. Scuseria, M. A. Robb, J. R. Cheeseman, G. Scalmani, V. Barone, G. A. Petersson, H. Nakatsuji, X. Li, M. Caricato, A. V. Marenich, J. Bloino, B. G. Janesko, R. Gomperts, B. Mennucci, H. P. Hratchian, J. V. Ortiz, A. F. Izmaylov, J. L. Sonnenberg, D. Williams-Young, F. Ding, F. Lipparini, F. Egidi, J. Goings, B. Peng, A. Petrone, T. Henderson, D. Ranasinghe, V. G. Zakrzewski, J. Gao, N. Rega, G. Zheng, W. Liang, M. Hada, M. Ehara, K. Toyota, R. Fukuda, J. Hasegawa, M. Ishida, T. Nakajima, Y. Honda, O. Kitao, H. Nakai, T. Vreven, K. Throssell, J. A. Montgomery, Jr., J. E. Peralta, F. Ogliaro, M. J. Bearpark, J. J. Heyd, E. N. Brothers, K. N. Kudin, V. N. Staroverov, T. A. Keith, R. Kobayashi, J. Normand, K. Raghavachari, A. P. Rendell, J. C. Burant, S. S. Iyengar, J. Tomasi, M. Cossi, J. M. Millam, M. Klene, C. Adamo, R. Cammi, J. W. Ochterski, R. L. Martin, K. Morokuma, O. Farkas, J. B. Foresman, and D. J. Fox, Gaussian, Inc., Wallingford CT, 2016.
4. Liu Z. Y., Lu T. & Chen Q.X. An sp-hybridized all-carboatomic ring, cyclo[18]carbon: Electronic structure, electronic spectrum, and optical nonlinearity, *Carbon* **165**, 461-467 (2020).
5. Lu, T, et al. Independent gradient model based on Hirshfeld partition: A new method for visual study of interactions in chemical systems, *Journal of computational chemistry* **43**, 539-555 (2022).
6. Lu, T, et al. Multiwfn: A multifunctional wavefunction analyzer. *Journal of computational chemistry* **33**, 580-592 (2012).
7. Lu, T. A comprehensive electron wavefunction analysis toolbox for chemists, Multiwfn. *The Journal of Chemical Physics* **161**, 082503 (2024).
8. Abraham, M. J. et al. GROMACS: High performance molecular simulations through multi-level parallelism from laptops to supercomputers. *SoftwareX* **1**, 19-25 (2015).
9. Wang, J. et al. Development and testing of a general amber force field. *Journal of computational chemistry* **25**, 1157-1174 (2004).

10. Feng, L. N. et al. Highly stable near-infrared II luminescent diradicaloids for cancer phototheranostics. *Journal of the American Chemical Society* **47**, 32582-32594 (2024).
11. You, C. F. et al. Strength in numbers: a giant NIR-II AIEgen with one-for-all phototheranostic features for exceptional orthotopic bladder cancer treatment. *Angewandte Chemie International Edition* e202417865 (2024).
12. Li, J. G. et al. As aggregation-induced emission meets with noncovalent conformational locks: subtly regulating NIR-II molecules for multimodal imaging-navigated synergistic therapies. *Angewandte Chemie International Edition* **64**, e202413219 (2024).
13. Li, Y. Y. et al. Incorporation of robust NIR-II fluorescence brightness and photothermal performance in a single large π -conjugated molecule for phototheranostics. *Advanced Science* **10**, 2204695 (2023).
14. Liu, C. C. et al. Furan donor for NIR-II molecular fluorophores with enhanced bioimaging performance. *Research* **6**, 39 (2023).
15. Liu, S. J. et al. Incorporation of planar blocks into twisted skeletons: boosting brightness of fluorophores for bioimaging beyond 1500 nanometer. *ACS Nano* **14**, 14228-14239 (2020).
16. Li, Y. X. et al. Promoted NIR-II fluorescence by heteroatom-inserted rigid-planar cores for monitoring cell therapy of acute lung injury. *Small* **18**, 2105362 (2022).
17. Zhang, Q. S. et al. Bright and stable NIR-II J-aggregated AIE dibodipy-based fluorescent probe for dynamic In vivo bioimaging. *Angewandte Chemie International Edition* **60**, 3967-3973 (2021).
18. Zhu, X. F. et al. High brightness NIR-II nanofluorophores based on fused-ring acceptor molecules. *Nano Research* **13**, 2570-2575 (2020).
19. Wang, S. F. et al. Anti-quenching NIR-II molecular fluorophores for in vivo high-contrast imaging and pH sensing. *Nature Communications* **10**, 1058 (2019).
20. Tian, R. et al. Rational design of a super-contrast NIR-II fluorophore affords high-performance NIR-II molecular imaging guided microsurgery. *Chemical Science* **10**, 326-332 (2019).
21. Yang, Q. L. et al. Donor engineering for NIR-II molecular fluorophores with enhanced

fluorescent performance. *Journal of the American Chemical Society* **140**, 1715-1724 (2018).

22. Wan, H. et al. Developing a bright NIR-II fluorophore with fast renal excretion and its application in molecular imaging of immune checkpoint PD-L1. *Advanced Functional Materials* **28**, 1804956 (2018).
23. Yang, Q. L. et al. Rational design of molecular fluorophores for biological imaging in the NIR-II window. *Advanced Materials* **29**, 1605497 (2017).
24. Li, C. B. et al. White-light activatable organic NIR-II luminescence nanomaterials for imaging-guided surgery. *Nature Communications* **15**, 5832 (2024).
25. Jiang, Q. H. et al. High-performance NIR-II fluorescent type I/II photosensitizer enabling augmented mild photothermal therapy of tumors by disrupting heat shock proteins. *Advanced Healthcare Materials.* **13**, 2400962 (2024).
26. Li, H. et al. Dimerization extends π-conjugation of electron donor-acceptor structures leading to phototheranostic properties beyond the sum of two monomers. *Aggregate* **5**, e528 (2024).
27. Wang, Y. W. et al. A NIR-II-emissive organic nanomedicine with biomimetic engineering for high-contrast targeted bioimaging and multiple phototherapies of pancreatic tumors. *Advanced Functional Materials* **34**, 2406483 (2024).
28. Gu, Y. et al. Chlorination-mediated π–π stacking enhances the photodynamic properties of a NIR-II emitting photosensitizer with extended conjugation. *Angewandte Chemie International Edition* **62**, e202303476 (2023).
29. Zhu, Y. L. et al. The balance effect of π–π electronic coupling on NIR-II emission and photodynamic properties of highly hydrophobic conjugated photosensitizers. *Advanced Science* **11**, 2307569 (2024).
30. Yin, B. L. *et al.* Tongue cancer tailored photosensitizers for NIR-II fluorescence imaging guided precise treatment. *Nano Today* **45**, 101550 (2022)
31. Zhu, Y. L. et al. Side-chain-tuned molecular packing allows concurrently boosted photoacoustic imaging and NIR-II fluorescence. *Angewandte Chemie International Edition* **61**, e202117433 (2022).

32. Li, C. B. et al. Fluorination enhances NIR-II emission and photothermal conversion efficiency of phototheranostic agents for imaging-guided cancer therapy. *Advanced Materials* **35**, 2208229 (2023).

33. Yuan, Y. et al. Molecular programming of NIR-IIb-emissive semiconducting small molecules for in vivo high-contrast bioimaging beyond 1500 nm. *Advanced Materials* **34**, 2201263 (2022).

34. Li, L. Q. et al. An NIR-II-emissive photosensitizer for hypoxia-tolerant photodynamic theranostics. *Advanced Materials* **32**, 2003471 (2020).
